# Highly stable and tunable peptoid/hemin enzymatic mimetics with natural peroxidase-like activities

**DOI:** 10.1038/s41467-022-30285-9

**Published:** 2022-05-31

**Authors:** Tengyue Jian, Yicheng Zhou, Peipei Wang, Wenchao Yang, Peng Mu, Xin Zhang, Xiao Zhang, Chun-Long Chen

**Affiliations:** 1 Physical Sciences Division, Pacific Northwest National Laboratory, Richland, WA 99352 USA; 2 The Voiland School of Chemical Engineering and Bioengineering, Washington State University, Richland, WA 99354 USA; 3 School of Chemical Engineering and Technology, State Key Laboratory of Chemical Engineering, Tianjin University, Tianjin, 300072 People’s Republic of China; 4 Department of Mechanical Engineering and Materials Science and Engineering Program, State University of New York, Binghamton, NY 13902 USA; 5 Department of Chemical Engineering, University of Washington, Seattle, WA 98195 USA

**Keywords:** Bioinspired materials, Molecular self-assembly, Self-assembly, Sustainability

## Abstract

Developing tunable and stable peroxidase mimetics with high catalytic efficiency provides a promising opportunity to improve and expand enzymatic catalysis in lignin depolymerization. A class of peptoid-based peroxidase mimetics with tunable catalytic activity and high stability is developed by constructing peptoids and hemins into self-assembled crystalline nanomaterials. By varying peptoid side chain chemistry to tailor the microenvironment of active sites, these self-assembled peptoid/hemin nanomaterials (Pep/hemin) exhibit highly modulable catalytic activities toward two lignin model substrates 2,2-azino-bis(3-ethylbenzothiazoline-6-sulfonic acid) and 3,3’,5,5’-tetramethylbenzidine. Among them, a Pep/hemin complex containing the pyridyl side chain showed the best catalytic efficiency (*V*_max_/*K*_m_ = 5.81 × 10^−3^ s^−1^). These Pep/hemin catalysts are highly stable; kinetics studies suggest that they follow a peroxidase-like mechanism. Moreover, they exhibit a high efficacy on depolymerization of a biorefinery lignin. Because Pep/hemin catalysts are highly robust and tunable, we expect that they offer tremendous opportunities for lignin valorization to high value products.

## Abstract (web summary)

Peroxidase mimics are currently being investigated as catalysts for lignin depolymerisation. In this article, the authors investigate a class of self-assembled and highly stable peptoid/hemin nanomaterials as peroxidase mimics that are highly stable and tuneable for the depolymerisation of a biorefinery lignin.

## Introduction

Peroxidases are one of the largest groups of oxidoreductases that catalyze the oxidation of many substrates mostly in the presence of H_2_O_2_, offering a wide variety of applications such as environmental protection, food processing, and clinical diagnostics^1^. Peroxidases have shown promising potentials for the valorization of renewable feedstock such as lignin, the largest renewable aromatics on earth^2^. For example, oxidative depolymerization of lignin into oligomers in the presence of peroxidase and H_2_O_2_ is a critical step in producing kerosene-type aviation fuels^3^. In these peroxidases, ferric center in hemin serves as an active site and the microenvironment around heme is surrounded by the polypeptide chains^4^. The cleavage of O–O bond of H_2_O_2_ occurs around the ferric center to form Fe^4+^=O and the hemin iron is stabilized in the catalytic cycle. In addition, the amino acid residues in the polypeptide chains surrounding the hemin active site, such as histidine (His), tryptophan (Trp), arginine (Arg), and glutamic acid (Glu), play a key role in regulating the peroxidase microenvironment^4^. Natural peroxidases are composed of proteins; thus, one cannot overlook the deficiencies of natural peroxidases such as the low stability under elevated temperatures, narrow optimal pH range, and susceptibility to denaturing. To circumvent these barriers, researchers have developed many hemin-containing peroxidase mimetics with enhanced stability for a broader range of applications^5,6^. These peroxidase mimetics include protein^7^, DNAzymes^8,9^, graphene^10^, graphene quantum dots^11^, metal-organic framework^12^, supramolecular polymers^13^, and inorganic nanoparticles^14^. Undoubtedly, these hemin-based peroxidase mimetics have significantly broadened the application conditions compared to natural peroxidases. Despite these advances, constructing highly efficient and robust peroxidase mimetics with natural enzyme-like flexibility in tuning active sites and microenvironments remains a grand challenge. It is therefore highly desirable to explore alternative materials that can mimic and tune the microenvironment of peroxidases while maintaining high stability under various conditions.

In this context, we developed a class of self-assembled peptoid/hemin (Pep/hemin) nanomaterials with tunable active sites and microenvironments that mimic peroxidases for lignin depolymerization, by taking advantage of the high tunability of peptoids (or poly-*N*-substituted glycines) and the uniqueness of their self-assembled crystalline nanomaterials in aligning active sites^15–18^. Compared to peptides, peptoids can be easily synthesized to achieve a greater side chain diversity while exhibiting much higher chemical and thermal stabilities^15–22^. Our recent work has shown that tuning amphiphilic peptoids can lead to the formation of hierarchically structured crystalline nanomaterials^17,18,23–28^, including membrane-mimetic 2D nanosheets^15,23,29^ and nanotubes^16,25,28^. These peptoid-based nanomaterials are highly stable in various pH conditions and at elevated temperatures. New functionalities and applications of peptoid nanomaterials can be easily realized by adjusting the side chain chemistry and by incorporating and aligning different functional groups^16,18,26,28^. Due to their high tunability and stability, peptoid nanomaterials are promising for creating optimal active sites and microenvironments. All these unique properties suggest that peptoid-based crystalline nanomaterials offer great opportunities for developing peroxidase mimetics.

Herein, a series of self-assembling peptoids with [2-(4-imidazolyl)ethylamine]glycine (Nhis), [2-(4-pyridyl)ethylamine]glycine (Npyr), or N-[2-(1H-indol-3-yl)ethyl]glycine (Ntrp) coordination sites were designed and co-assembled with hemin to form crystalline nanomaterials. The catalytic activity of these peroxidase mimetics were evaluated using two representative model compounds: 2,2-azino-bis(3-ethylbenzothiazoline-6-sulfonic acid) (ABTS) and 3,3’,5,5’-tetramethylbenzidine (TMB) that are commonly used to evaluate the oxidative catalytic capability of peroxidases. We found that peptoid-based mimetics containing Npyr groups showed the highest catalytic capability. By further varying the side chain chemistry of Npyr-containing peptoids, we demonstrated the tuning of hemin-Npyr microenvironments of these peroxidase mimetics. Our results showed those with N-(2-carboxyethyl)glycine (Nce) groups and optimized distance between Npyr and the hydrophobic domain exhibited the best catalytic efficiency with a *V*_max_ /*K*_m_ value of 5.81 × 10^−3^ s^−1^ at 0.079 mg/ml catalyst concentration, which is among the highest activities that have been previously reported for hemin-containing peroxidase mimetics^10–12,30,31^. By testing the oxidation activity of Pep/hemin at 60 and 90 °C, we demonstrated that they remained active and increased reaction rates under elevated temperatures. Three most promising Pep/hemin mimetics were further tested for their high efficacy in the depolymerization of organosolv lignin obtained fromlodgepole pine. Our results showed that these peptoid-based enzyme mimetics can catalyze the oxidative depolymerization of organosolv lignin under much milder conditions with a shorter incubation time in contrast to peroxidase-based enzymic lignin depolymerization^2,32^.

## Results

### Design of ligand-containing peptoids

**Fig. 1:**
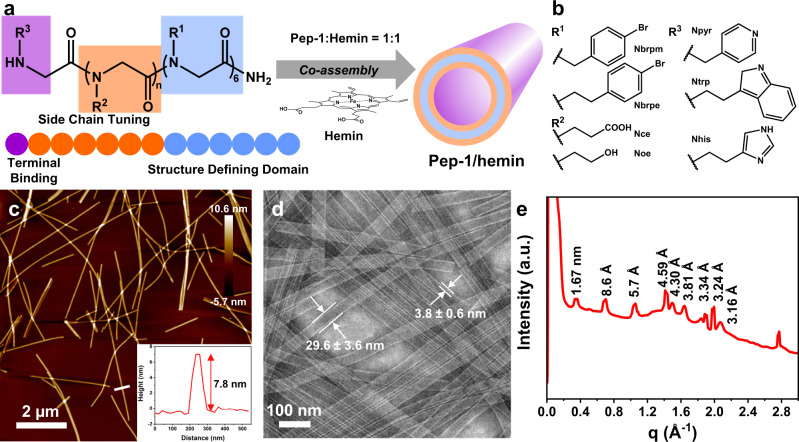
Peptoid sequences and their assembled nanotube structures. **a** Molecular and schematic representations of peptoid sequences and its co-assembly with hemin into nanotube morphology. **b** Side chain and terminal modifications of peptoid sequences. **c** AFM image of showing the co-assembled nanotube structure of **Pep-1** (R^1^ = Nbrpm, R^2^ = Nce, R^3^ = Npyr, *n* = 6) and hemin. The inset shows the height the nanotube is around 7.8 nm. **d** TEM image showing the **Pep-1/hemin** nanotube with a width of 29.6 ± 3.6 nm and a wall thickness of 3.8 ± 0.6 nm based on at least 50 counts. **e** XRD spectrum of **Pep-1/hemin** nanotube. The value above each peak is calculated based on the equation *d* = 2*π*/*q*.

To mimic the catalytic site of natural peroxidases and take advantage of the coordination of hemin with self-assembling peptoids, a series of peptoids with various coordination sites (i.e., Npyr, Nhis, or Ntrp) are designed and synthesized: Nbrpm_*n*_Nce_*m*_-R (*n* = 6, *m* = 0–9, R = Npyr, Nhis, or Ntrp; Nbrpm = N-[(4-bromophenyl)methyl]glycine), as shown in Fig. 1a, b. We chose Npyr, Nhis, or Ntrp as terminal ligands because these functional groups are found to facilitate the stabilization of hemin in the active site of natural peroxidases^11^. Because the local environment of hemin active sites within natural peroxidases is important to facilitate lignin depolymerization, and amino acid residues, such as Asp and Phe surrounding hemin, facilitate the catalytic activity via carboxylate groups and hydrophobic interactions^11^, we further designed and synthesized a number of peptoid sequences with variations in coordination sites, the length of polar side-chain domain, and the side chain chemistry.

### Assembly and characterizations of Pep-1/hemin nanotubes

To generate crystalline nanotubes with stabilized hemins as active sites, tube-forming peptoid **Pep-1** containing Nbrpm_6_, Nce_6_, and Npyr (Fig. 1a) was synthesized. **Pep-1/hemin** nanotube was prepared by mixing and incubating **Pep-1** with equimolar hemin similar to our previously reported method^16^. Briefly, lyophilized **Pep-1** was incubated with hemin in water and acetonitrile (v/v = 50:50) at 4 °C for slow crystallization. Gel-like materials containing a large amount of crystalline peptoid nanotubes formed after 2 to 3 days of incubation (see Methods for details). Atomic force microscopy (AFM) studies showed that the co-assembled **Pep-1/hemin** materials exhibited a well-defined tubular structure (Fig. 1c) with an average tube height of 7.8 nm, which is similar to the data we previously reported for deformed nanotubes under dry condition^16^. Transmission electron microscopy (TEM) also revealed that the diameter of the nanotube is 29.6 ± 3.6 nm, and the wall thickness of the nanotube is 3.8 ± 0.6 nm (Fig. 1d and Supplementary Fig. 1). **Pep-1/hemin** nanotubes observed in both AFM and TEM images are similar to our previous results^16^. To further examine the structure of the **Pep-1/hemin** assembly, we performed X-ray diffraction (XRD) measurement of self-assembled **Pep-1/hemin** nanotubes. XRD data showed that **Pep-1/hemin** nanotubes are highly crystalline and exhibit a similar structure to our previously reported peptoid nanotubes (Fig. 1e). The peak 1.67 nm corresponds to the distance between two peptoid backbones and the peak at 5.7 Å corresponds to the ordered packing of aromatic side chains within the hydrophobic domain. The strong peak at 4.59 Å is from the space of the alignment of peptoid chains between peptoids. The peaks at 4.30 and 3.81 Å can be ascribed to the presence of *π*-*π* interaction^33–35^.

Having confirmed the assembly morphology and crystallinity of the **Pep-1/hemin**, we continued to use ultraviolet-visible (UV-vis) absorption spectroscopy to determine the optimal assembly ratio between **Pep-1** and hemin. An absorption peak at 503 nm, indicating the coordination of **Pep-1** and hemin, increases as hemin is titrated into a solution of **Pep-1** (Supplementary Fig. 2a)^36^. The rise of the peak plateaus at 1.0 mol of hemin for 1.0 mol of **Pep-1** (Supplementary Fig. 2b), suggesting a 1:1 ratio of **Pep-1** and hemin for coordination.

### Peroxidase-like activity in ABTS oxidation

**Fig. 2:**
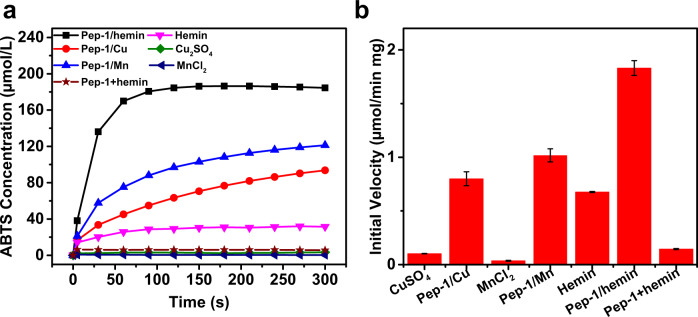
Peroxidase mimetic activity of Pep-1/hemin, its constituent components, and its metal substitutes. **a** Change in ABTS concentration by H_2_O_2_ over time in the presence of **Pep-1/hemin** complex, simple mixing of **Pep-1** nanotubes and hemin, **Pep-1/Cu** complex, **Pep-1/Mn** complex, free hemin, CuSO_4_ salt, and MnCl_2_ salt. **b** Initial velocities of reactions catalyzed by CuSO_4_, **Pep-1/Cu**, MnCl_2_, **Pep-1/Mn**, hemin, **Pep-1/hemin**, and simple mixing of Pep-1 nanotubes and hemin (namely **Pep-1** + **hemin**). The error bar represents the standard deviation of three measurements.

To test the peroxidase-like activity for potential lignin depolymerization, we used self-assembled **Pep-1/hemin** for oxidation of ABTS, a commonly used model substrate to reveal peroxidase-like catalytic properties in the presence of H_2_O_2_. As shown in Fig. 2a (black), the concentration of oxidized ABTS increases quickly in the first 90 s upon reacting with self-assembled **Pep-1/hemin** nanotubes. The initial velocity of **Pep-1/hemin** nanotubes toward the oxidation of ABTS is 1.862 ± 0.069 μmol min^−1^ mg^−1^ (see “Methods” for detail).

To demonstrate the influence of nanotube metal centers on the oxidation of ABTS, we further prepared nanotubes by self-assembly of **Pep-1** in the presence of Cu^2+^ or Mn^2+^ (see “Methods” for details), with the intention of using these metal cations to mimic the hemin active sites of natural peroxidase enzymes^37,38^. The ensuing tubular complexes, namely **Pep-1/Cu** and **Pep-1/Mn**, were prepared in a similar way to **Pep-1/hemin** (see “Methods” for experimental details). AFM images of **Pep-1/Cu** and **Pep-1/Mn** showed well-defined nanotube structures with average heights of 8.3 ± 0.4 nm and 7.9 ± 0.6 nm, respectively (Supplementary Fig. 3). XRD patterns of **Pep-1/Cu** and **Pep-1/Mn** also revealed that both assembled nanotubes are highly crystalline (Supplementary Fig. 4) and exhibit a similar tubular structure to those of **Pep-1/hemin** and **Pep-1** nanotube. The nanotube structures of **Pep-1/hemin**, **Pep-1/Cu**, and **Pep-1/Mn** were further confirmed by scanning electron microscopy (SEM) imaging. Energy-dispersive X-ray spectroscopy (EDS) measurements confirmed the presence of corresponding metal cations bound on nanotubes (Supplementary Fig. 5), indicating these metal cations are complexed into the nanotubes. As shown in Fig. 2a, under the same reaction conditions, ABTS absorbances with both **Pep-1/Cu** and **Pep-1/Mn** nanotubes showed significantly slower increases compared to that with **Pep-1/hemin** nanotubes. Specifically, the initial velocities of **Pep-1/Cu** (0.800 ± 0.065 μmol min^−1^ mg^−1^) and **Pep-1/Mn** (0.036 ± 0.004 μmol min^−1^ mg^−1^) complexes (Fig. 2b) were significantly smaller. These results show that the type of metal centers of self-assembled peptoid nanotubes are critical to ABTS oxidation. Among them, those with well-organized hemins showed the best activity. To further confirm the importance of high ordering of hemins in the ABTS oxidation, we prepared hemin-containing nanotubes by simply mixing the pre-formed **Pep-1** nanotubes with equimolar hemin, we referred to these hemin-containing tubes as **Pep-1** + **hemin**. The ABTS oxidation results showed that this **Pep-1** + **hemin** sample only led to a poor performance with an initial velocity (*V*_0_) of 0.145 ± 0.005 μmol min^−1^ mg^−1^, further confirming the importance of hemin ordering in achieving high peroxidase-like activity.

### Tunability of peptoid nanomaterials in ABTS oxidation

To demonstrate the feasibility of peptoid nanotubes as tunable peroxidase mimetics with tunable active sites and microenvironments, we systematically varied the coordinating terminal ligand, the length of polar side chain domain, the side chain chemistry, and the morphology of self-assembled nanomaterials. Initial velocity was measured for these nanomaterials to evaluate their activities in ABTS oxidation. The detailed synthesis and characterizations of these peptoids and peptoid nanomaterials are provided in Methods and Supplementary Figs. 6–24.

#### Terminal groups

Because the interaction of imidazolyl group with the ferric center of natural peroxidase enzymes is critical for the catalytic activity^4,11^, to mimic such interaction and create active sites, peptoids **Pep-2** and **Pep-3** with two different coordinating terminal ligands (Ntrp and Nhis) and **Pep-4** without a terminal coordinating ligand were used for the self-assembly of nanomaterials as peroxidase mimetics. AFM results showed that all peptoids (**Pep-2** to **Pep-4**) formed nanotubes by co-assembly with hemin (Supplementary Figs. 16–18). The observed similar heights suggest that all nanotubes show a similar structure to **Pep-1/hemin** nanotubes. Results on ABTS oxidation showed that both **Pep-2/hemin** and **Pep-3/hemin** nanotubes showed a slightly decreased catalytic activity compared to **Pep-1/hemin**. Specifically, **Pep-2/hemin** with an Ntrp ligand shows an initial velocity of 1.413 ± 0.081 μmol min^−1^ mg^−1^, while **Pep-3/hemin** with Nhis group shows an initial velocity of 1.502 ± 0.120 μmol min^−1^ mg^−1^. Compared to **Pep-1/hemin**, **Pep-2/hemin** and **Pep-3/hemin**, nanotubes assembled from **Pep-4** showed an initial velocity of 0.437 ± 0.023 μmol min^−1^ mg^−1^, suggesting the importance of terminal ligands in the creation of active sites to achieve high catalytic efficiency. Among the three terminal ligands (i.e., Nhis, Npyr, Ntrp), nanotubes containing Npyr (i.e., **Pep-1/hemin**) showed the highest catalytic activity in ABTS oxidation which could be due to the strongest coordination of Npyr with hemin^39,40^.

#### Backbone length

In the natural peroxidase, the active center of hemin is usually located in a hydrophobic environment, which is critical for achieving high catalytic activity^4^. To demonstrate that the distance between the catalytic sites and the hydrophobic domains is important for peroxidase-like activity, we obtained similar nanotubes (Supplementary Figs. 19–22) using peptoids (**Pep-5** to **Pep-8**) with varied number (*n*) of Nce groups (from 0 to 9). ABTS oxidation results showed that the initial velocities of these nanotubes reached the maximum when the number of Nce group is 5 (**Pep-7**) or 6 (**Pep-1**); nanotubes assembled from either a low *n* (**Pep-5**, *n* = 0; **Pep-6**, *n* = 3) or a high *n* (**Pep-8**, *n* = 9) showed a decreased initial velocity. These results suggest that having an optimal distance between the catalytic sites and the hydrophobic domain is important to achieve a high efficiency. We speculate that by changing the distance between the coordinating terminal ligand and hydrophobic domain of peptoids, the active sites composed of hemin and Npyr are located at the optimal distance from the hydrophobic cores of assembled nanotubes, thus offering the optimal hydrophobic environment for interactions with the ABTS substrate.

#### Side chain chemistry

The active site in the natural peroxidase is often stabilized by various functional groups in the surrounding amino acid residues, such as the -COOH group from Asp^1,41^. To demonstrate the importance of -COOH group in the peptoid/hemin nanotube to mimic peroxidase, we further prepared similar nanotubes using **Pep-9** that has six N-(2-hydroxyethyl)glycine (Noe) groups as the polar side chains instead of Nce groups while keeping the same Npyr coordinating terminal ligand and hydrophobic domain (Supplementary Fig. 23). ABTS oxidation results showed that these **Pep-9/hemin** nanotubes exhibited a significant decrease in catalytic activity. Compared with **Pep-1**, we reasoned that **Pep-9** with six Noe groups is unable to facilitate the proton transfer and thus leads to reduced efficiency in oxidation reaction^11,42^.

#### Assembly morphology

The morphology of self-assembled enzyme mimetic can be significant for influencing its catalytic activity. In fact, various morphologies of self-assembled materials have been designed to mimic the catalytic activity of natural enzymes^43–45^. To demonstrate the importance of tubular structure in the oxidation of ABTS, we synthesized **Pep-10** by replacing Nbrpm_6_ in the hydrophobic domain with Nbrpe_6_ (Nbrpe = N-[2-(4-bromophenyl)ethyl]glycine). Co-assembly of this peptoid with hemin led to the formation of a nanosheet structure (**Pep-10/hemin**, Supplementary Figs. 24 and 25), which is similar to our previously reported membrane-mimetic nanosheets^24^. ABTS oxidation results showed that **Pep-10/hemin** nanosheets showed a slight decrease in catalytic activity with an initial velocity of 1.542 ± 0.019 µmol min^−1^ mg^−1^, suggesting the overall morphology of self-assembled crystalline nanomaterials can impact the catalytic activity. Further optimization of nanosheet catalysts is now underway.

### High stability of biomimetic catalysts

**Fig. 3:**
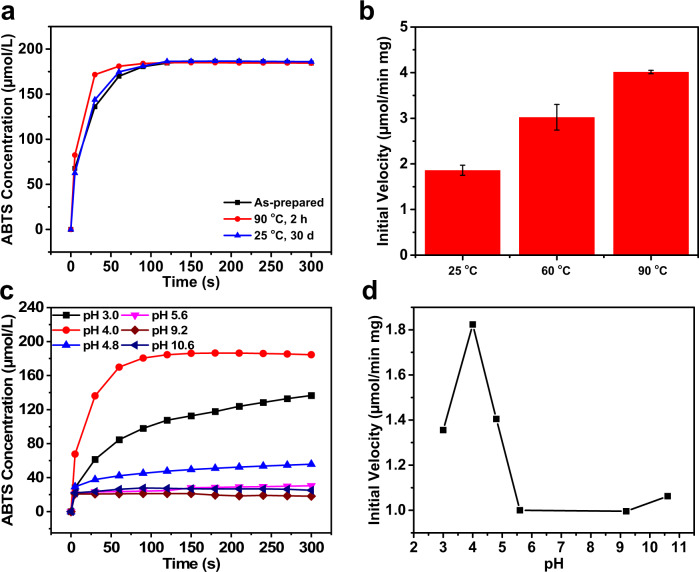
Peroxidase activity of Pep-1/hemin as a function of temperature and solution pH. **a** Change in ABTS concentration by H_2_O_2_ over time in the presence of **Pep-1/hemin** (black), **Pep-1/hemin** after incubated at 90 °C for 2 h (red), and **Pep-1/hemin** after incubated at room temperature for 30 days (blue). **b** Initial velocities of ABTS oxidation reactions in the presence of **Pep-1/hemin** after it has been incubated at 25, 60, and 90 °C for 2 h. The error bar represents the standard deviation of three measurements. **c** Change in concentration of ABTS with H_2_O_2_ over time in the presence of **Pep-1/hemin** at different pHs. **d** Initial velocities of ABTS oxidation reactions in the presence of **Pep-1/hemin** at different pHs.

One of the main motivations for the development of peroxidase mimetics is to enable peroxidase-like reactions to perform under harsh conditions in which natural enzymes cannot survive. Therefore, the activities of these tubular Pep/hemin nanomaterials on ABTS oxidation were determined at elevated temperatures and different solution pHs. Figure 3a showed time-dependent ABTS oxidation under room temperature by **Pep-1/hemin**, by **Pep-1/hemin** after 30-day room temperature incubation, or by **Pep-1/hemin** that had been treated under 90 °C for 2 h. It is clear that neither high temperature treatment (i.e., 90 °C for 2 h) nor 30-day incubation of **Pep-1/hemin** reduces its activity. To further evaluate the operational thermostability of **Pep-1/hemin**, we next examined **Pep-1/hemin** activities at 60 and 90 °C. In this experiment, the **Pep-1/hemin** nanotubes were first incubated at 60 °C or 90 °C for 2 h. Immediately after the incubation, preheated ABTS solutions (to 60 °C or 90 °C) were added and the initial reaction velocities were determined (Fig. 3b). It is apparent that higher temperature favors the ABTS oxidation rate induced by **Pep-1/hemin**. **Pep-1/hemin** showed higher activity at 60 °C or 90 °C than at 25 °C. In contrast, natural lignin peroxidases have poor thermostability and are typically denatured at 60 °C^46^. The ability of **Pep-1/hemin** to remain active and increase reaction rate under elevated temperatures is certainly a significant advantage over natural lignin peroxidases. The high stability of **Pep-1/hemin** was further confirmed for the ABTS oxidation of **Pep-1/hemin** under various solution pHs (from 3.0 to 10.0) (Fig. 3c, d). As demonstrated by the initial velocity measurements, the maximum activity of **Pep-1/hemin** is reached at an acidic pH of 4, which is similar to the optimal pH for natural peroxidases^11^. To further confirm these **Pep-1/hemin** nanotubes are highly stable, we examined the morphology of **Pep-1/hemin** after ABTS oxidation reactions under 60 and 90 °C. TEM studies showed that self-assembled **Pep-1/hemin** remained the tubular morphology (Supplementary Fig. 26). All these findings showed that **Pep-1/hemin** enzyme mimetics are highly robust and remain active in a variety of conditions, i.e., elevated temperatures and solution pH from 3.0 to 10.0.

### Kinetic investigation of the mechanism of Pep-1/hemin reaction with ABTS and H_2_O_2_

To react with the substrate, peroxidases commonly require the presence of H_2_O_2_. In bi-substrate enzyme reactions, there are two main kinetic mechanisms: sequential mechanism (ternary complex formation) and ping-pong mechanism (double replacement reaction). The initial velocity (*V*_0_) equation for bi-substrate (ABTS and H_2_O_2_) can be expressed in Dalziel’s form (Eq. (1)). The reciprocal form of the initial velocity equation is shown in Eq. (2) which can be modified to Eq. (3). *θ* is the reciprocal coefficient expressed in Dalziel form or Dalziel coefficient^47^, and *θ*_12_ is associated with both substrates. A significant *θ*_12_ value confirms the formation of a ternary complex among the enzyme with both substrates whereas a negligible *θ*_12_ value suggests a double replacement ping-pong mechanism^48^.

1 $${v}_{0}=\frac{\left[{{{{{\rm{H}}}}}}_{2}{{{{{\rm{O}}}}}}_{2}\right][{{{{{{\rm{ABTS}}}}}}}]}{{\theta }_{12}+{\theta }_{2}[{{{{{\rm{{H}}}}}}}_{2}{{{{{\rm{{O}}}}}}}_{2}]+{\theta }_{1}\left[{{{{{{\rm{ABTS}}}}}}}\right]+{\theta }_{0}\left[{{{{{{\rm{H}}}}}}}_{2}{{{{{\rm{{O}}}}}}}_{2}\right][{{{{{{\rm{ABTS}}}}}}}]}$$

2 $$\frac{1}{{v}_{0}}={\theta }_{0}+\frac{{\theta }_{1}}{[{{{{{{\rm{ABTS}}}}}}}]}+\frac{{\theta }_{2}}{[{{{{{\rm{{H}}}}}}}_{2}{{{{{\rm{{O}}}}}}}_{2}]}+\frac{{\theta }_{12}}{[{{{{{{\rm{ABTS}}}}}}}][{{{{{{\rm{H}}}}}}}_{2}{{{{{{\rm{O}}}}}}}_{2}]}$$

3 $$\frac{1}{{v}_{0}}=\left({\theta }_{0}+\frac{{\theta }_{2}}{[{{{{{{\rm{H}}}}}}}_{2}{{{{{{\rm{O}}}}}}}_{2}]}\right)+\left({\theta }_{1}+\frac{{\theta }_{12}}{[{{{{{{\rm{H}}}}}}}_{2}{{{{{{\rm{O}}}}}}}_{2}]}\right)\frac{1}{[{{{{{{\rm{ABTS}}}}}}}]}$$

**Fig. 4:**
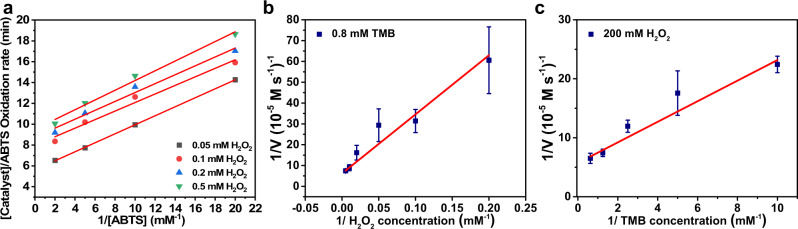
Kinetic studies of Pep-1/hemin. **a** Lineweaver-Burk linear fit with variation of ABTS concentrations under four different H_2_O_2_ dosages. Lineweaver-Burk activity for **Pep-1/hemin** at a fixed concentration of one substrate, 0.8 mM TMB for **b** or 200 mM H_2_O_2_ for **c** versus varying the concentration of a second substrate (H_2_O_2_ for **b** or TMB for **c**). The *V* or each data point is the concentration variation of the reaction system per second. The error bar represents the standard deviation of three measurements. Red line is the linear fit for each experiment.

To investigate the kinetic mechanism of **Pep-1/hemin** oxidation of ATBS in the presence of H_2_O_2_, the initial ABTS oxidation rates were determined at four constant H_2_O_2_ dosages, 0.05, 0.1, 0.2, and 0.5 mM, each with a variable amount of ABTS concentrations (as arranged in Eq. (3)). As shown in Fig. 4a, the four Lineweaver-Burk plots obtained from each constant H_2_O_2_ dosage with various amounts of ABTS appear to be parallel to each other, suggesting that the oxidation of ABTS induced by **Pep-1/hemin** follows a ping-pong mechanism. The secondary plot, which is obtained using the slope values from the four Lineweaver-Burk plots (${\theta }_{1}+\frac{{\theta }_{12}}{[{{{{{\rm{{H}}}}}}}_{2}{{{{{\rm{{O}}}}}}}_{2}]}$) against inversed H_2_O_2_ changes (Supplementary Fig. 27), showed a linear fitting with a very small slope (0.00148) correlating to a negligible *θ*_12_ value, verifying the double replacement ping-pong mechanism.

To further evaluate the catalytic performance of the Pep/hemin complexes compared to some existing peroxidase mimetics^5,10–12,30,31^, the enzymic kinetic parameters, including Michaelis-Menten constant (*K*_m_), maximum initial velocity (*V*_max_), and *V*_max_/*K*_m_ ratio, were obtained on another substrate, 3,3’,5,5’-tetramethylbenzidine (TMB). Similarly, typical Michaelis-Menten curves were plotted by measuring the initial velocity at different concentrations of TMB (0.4, 0.8, and 1.6 mM) and H_2_O_2_ (50, 100, and 200 mM). Both *K*_m_ and *V*_max_ values were obtained by fitting the data to the Lineweaver-Burk equation (Fig. 4b, c). For **Pep-1/hemin**, the *K*_m_ value of **Pep-1/hemin** for TMB is 0.264 mM, and the *V*_max_ value of **Pep-1/hemin** for TMB is 1.54 × 10^−6^ M s^−1^. Consequently, the *V*_max_/*K*_m_ value, a common measure to evaluate the catalytic efficiency, is calculated at 5.81 × 10^−3^ s^−1^, which is one order of magnitude higher than the most active hemin-containing peroxidase mimetics reported so far (Table 1)^5,10–12,30,31^. The results indicate that our best Pep/hemin catalyst displays a highly efficient performance amongst the reported hemin-containing peroxidase mimetics at yet a similar mass concentration (0.079 mg/ml).

**Table 1:** Comparison of kinetic parameters of Pep-1/hemin and other hemin-containing peroxidase mimetics.

Catalyst	Substrate	*K*_*m*_ (mM)	*V*_max_ (×10^−8^ M s^−1^)	*V*_max_/*K*_*m*_ (×10^−5^ s^−1^)
Pep-1/hemin	TMB H_2_O_2_	0.264 28.3	153.5 109.8	581.4 3.88
Hemin^10^	TMB	4.84	4.69	0.969
His-GQD/hemin^11^	TMB H_2_O_2_	0.133 3.8	9.7 10.55	72.9 2.77
Hemin-Graphene^10^	TMB	5.1	4.55	0.89
Hemin@MOF^12^	TMB	0.068	6.07	89.26
Hemin-WS_2_^30^	TMB	0.467	6.45	13.81
Hemin/ZIF-8^31^	TMB	0.024	0.91	37.91

### Reaction mechanism

**Fig. 5:**
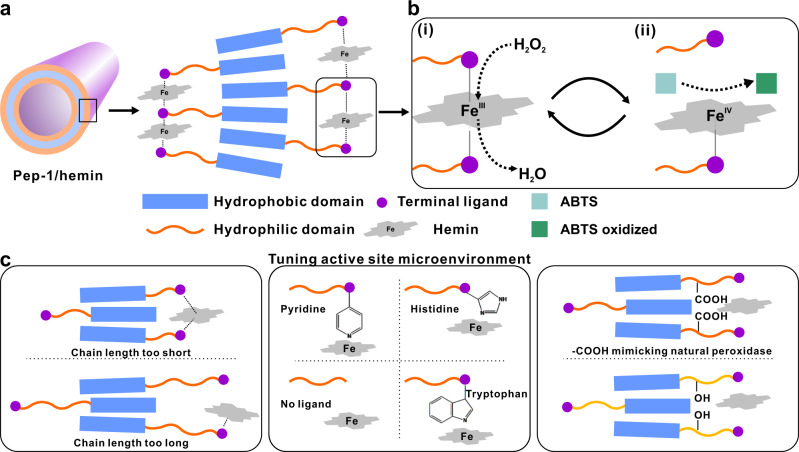
Schematic representation of systematic tuning of peptoid/hemin assemblies toward their catalytic activities and a proposed reaction mechanism. **a** **Pep-1/hemin** nanotube and the proposed assembly mechanism. **b** Catalytic mechanism showing the ping-pong pathway for the oxidation of ABTS. **c** Tuning microenvironments of peptoid/hemin catalyst in terms of chain length, terminal ligand, and side chain.

Based on the results, we propose that the oxidation of ABTS substrates in the presence of **Pep-1/hemin** and H_2_O_2_ follows a pathway as shown in Fig. 5. Since the reaction follows a ping-pong pathway, an optimized microenvironment for the active site of Pep/hemin catalyst is critical for ABTS oxidation. In a typical **Pep-1/hemin** catalyst (Fig. 5a), the hydrophobic domain of peptoids is packed and embedded in the center of the tubular wall, while the hydrophilic domain is extending to the surface of the wall. Conjugating a hemin-binding ligand at the terminus of the hydrophilic domain enables the immobilization of hemins onto the peptoid nanotube. As the coordination between hemin and terminal coordination ligand occurs, self-assembled Pep/hemin nanotubes can have natural peroxidase-like microenvironments. As discussed above, the reaction mechanism follows a ping-pong model, i.e., the two substrates, H_2_O_2_ and ABTS, sequentially react with the metal center, where H_2_O_2_ oxidizes the Fe^III^ to Fe^IV^ (step-i in Fig. 5b), followed by oxidation of the other substrate ABTS (step-ii in Fig. 5b). Consequently, when the chemical structure of a peptoid is systematically varied, the overall catalytic activity of the Pep/hemin complex can be tuned. For example, the length of the polar domain plays a key role in the catalytic activity of peptoid/hemin for ABTS oxidation (Fig. 5c, left panel). We speculate that shorter hydrophilic domain (Nce_3_) constrains the coordination between the terminal ligand and hemin, thus resulting in weaker coordination between peptoid and hemin. Longer hydrophilic domain (Nce_9_), on the other hand, may be too flexible for strong coordination to take place. In addition, hemin is distanced from the hydrophobic domain by the long peptoid chain, whereas, in a natural peroxidase, the active site is encased in a hydrophobic environment. Thus, an optimized chain length (Nce_6_) is needed for improving catalytic activity. Furthermore, we chose to use Nce groups to mimic amino acid residues like Asp in the polypeptide chain surrounding the active site in a natural peroxidase (Fig. 5c, center panel)^49^. As our results suggest, Pep/hemin nanotube with Nce groups in the side chain close to the terminus showed higher activity than that with Noe groups. Finally, in terms of assembly morphology, we speculate that the difference in catalytic activity may be attributed to the unique curvature of nanotube surfaces, in which hemins are more exposed than those on the sheet surfaces for interacting with ABTS substrate or H_2_O_2_ molecule (Fig. 5c, right panel). All these results demonstrate that the Pep/hemin catalysts are highly tunable and versatile in mimicking the microenvironment of the active site in the natural peroxidase.

### Lignin depolymerization by peptoid/hemin catalysts

Although two major lignin peroxidases, lignin peroxidase (LiP, EC 1.11.1.14) and manganese peroxidase (MnP, EC 1.11.1.13), were discovered almost 40 years ago^50–53^, practical application of peroxidase for lignin depolymerization remains a long-term objective. While many LiP and MnP enzymes have shown high activities against lignin model substrates, including ABTS, veratryl alcohol, and syringaldazine (SGZ), in the presence of H_2_O_2_, there is very little experimental evidence demonstrating that these enzymes can depolymerize real lignin samples^2^. In fact, when peroxidase/H_2_O_2_ systems were tested on bulk lignin samples, polymerization instead of depolymerization was often observed as the overall reaction outcome^54,55^. The size of the lignin substrate is a key factor affecting its activity with LiP/H_2_O_2_. Baciocchi has shown that the catalytic efficiency of LiP/H_2_O_2_ oxidizing a lignin trimer model compound is only a fraction of a monomeric model compound, despite both substrates showing a summarily reactivity to a chemical oxidant^56^. LiP and MnP are biomacromolecules with a molecular weight (MW) of over 38 kDa. Lignin is also a macromolecule that is assembled by multiple phenylpropane subunits. It is conceivable that a single-active-site-containing peroxidase has a significant challenge to effectively catalyze the bond cleavage in bulk lignin substrate^2,57^.

**Fig. 6:**
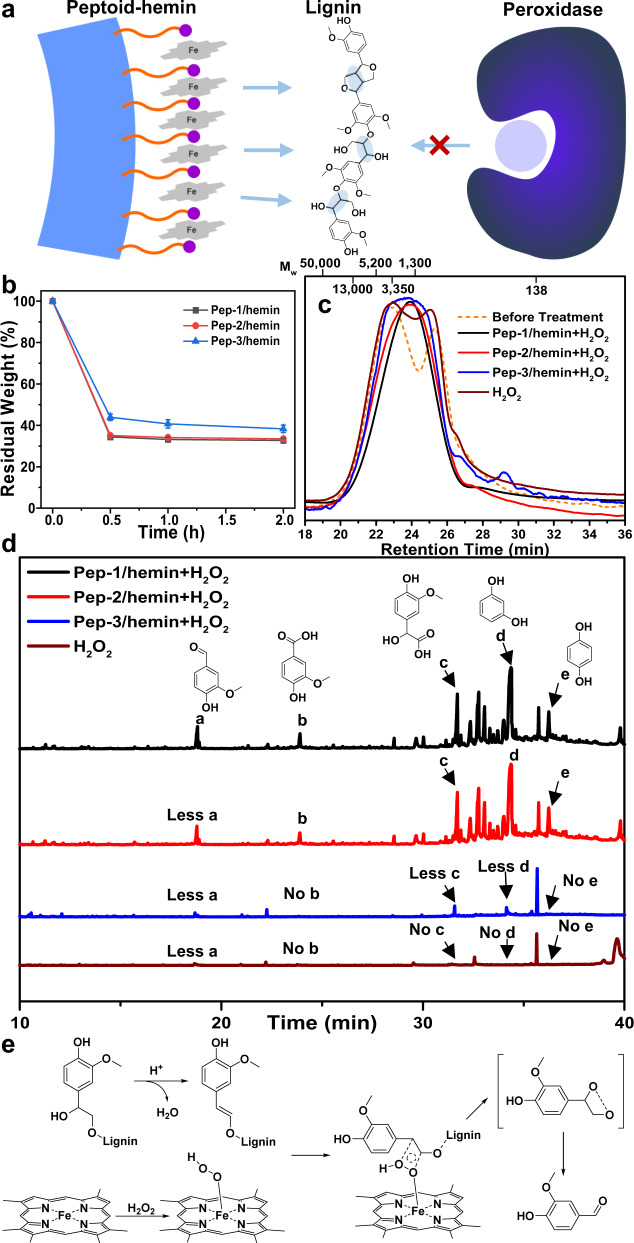
Lignin depolymerization by Pep/hemin nanomaterial catalysts. **a** Schematic representation showing the multiple active sites on peptoid-hemin nanomaterial interacting with lignin (left) compared to a single-active site on lignin peroxidase (right). **b** Residual weight percent of EOL after treated with **Pep-1/hemin**, **Pep-2/hemin**, and **Pep-3/hemin** for 30 min, 1 h, and 2 h. The error bar represents the standard deviation of three measurements. **c** GPC profile of EOL samples before treatment, after treatment with **Pep-1/hemin**, **Pep-2/hemin**, or **Pep-3/hemin** in the presence of H_2_O_2_, after treatment only with H_2_O_2_. **d** GC-MS results of ethyl acetate extractable products, including five phenolic compounds a–e, from lodgepole pine lignin treated with or without peptoid/hemin catalysts at 60 °C under acidic solution (pH~4) in the presence of H_2_O_2_. **e** Proposed reaction mechanism of oxidative depolymerization of lignin with the heme center in peptoid/hemin nanomaterial catalyst.

Due to the unique structural features and high efficacy in ABTS oxidization, Pep/hemin catalysts are promising to overcome the barrier of tuning enzyme-substrate interactions. As illustrated in Fig. 6a, a single Pep/hemin nanotube carries multiple highly ordered active sites which can simultaneously oxidize multiple reactive sites in lignin to promote effective depolymerization. These active sites are exposed on the Pep/hemin surface which minimizes the steric hindrance and electron transfer issues that are inherited to peroxidases^2,58,59^. Therefore, we reason that peptoid/hemin complexes with high efficiency in ATBS oxidation are suitable for efficient depolymerization of lignin polymers. To test the feasibility of Pep/hemin complexes in depolymerization of lignin, three Pep/hemin complexes, **Pep-1/hemin**, **Pep-2/hemin**, and **Pep-3/hemin**, which have shown significant activities in ABTS oxidation (Table 2), were used to depolymerize a biorefinery lignin sample, ethanol organosolv lignin (EOL) obtained from organosolv pretreatment lodgepole pine (*Pinus contorta*)^60,61^, in the presence of H_2_O_2_. The EOL represents a lignin polymer from softwood with an *M*_n_ around 1470 and an *M*_w_ around 4170. Treatments of EOL were conducted at 60 °C under an acidic solution (pH ~4) in the presence of H_2_O_2_. It was surprising to find that after 30 min of reaction, most of EOL were depolymerized and dissolved in the aqueous solution (See discussions below). The residual EOL solids were collected and analyzed via gel permeation chromatography (GPC) (see “Methods” for experimental details). The depolymerization products in the reaction solution were extracted by ethyl acetate and subjected to gas chromatography-mass spectroscopy (GC-MS) analysis after silylation.

**Table 2:** Peptoids used for constructing peptoid/hemin peroxidase mimetics with mutations in sequence and the corresponding initial velocities.

Peptoid nanomaterial	Sequence	Variation	Initial velocity (*V*_0_) (μmol min^−1^ mg^−1^)
**Pep-1/hemin**	Nbrpm_6_Nce_6_Npyr	N/A	1.862 ± 0.069
**Pep-1** + **hemin**	Nbrpm_6_Nce_6_Npyr		0.145 ± 0.005
**Pep-2/hemin**	Nbrpm_6_Nce_6_Ntrp	Terminal group	1.413 ± 0.081
**Pep-3/hemin**	Nbrpm_6_Nce_6_Nhis		1.502 ± 0.120
**Pep-4/hemin**	Nbrpm_6_Nce_6_		0.437 ± 0.023
**Pep-5/hemin**	Nbrpm_6_Npyr	Backbone length	0.956 ± 0.036
**Pep-6/hemin**	Nbrpm_6_Nce_3_Npyr		0.970 ± 0.039
**Pep-7/hemin**	Nbrpm_6_Nce_5_Npyr		1.693 ± 0.038
**Pep-8/hemin**	Nbrpm_6_Nce_9_Npyr		0.900 ± 0.040
**Pep-9/hemin**	Nbrpm_6_Noe_6_Npyr	Side chain	0.804 ± 0.051
**Pep-10/hemin**	Nbrpe_6_Nce_6_Npyr	Assembly morphology	1.542 ± 0.019

As shown in Fig. 6b and Supplementary Table 1, the weights of residual EOL after respective **Pep-1/hemin**, **Pep-2/hemin**, **Pep-3/hemin**, and no Pep/hemin catalyst treatments in the presence of H_2_O_2_ are 34.3%, 35.1%, and 43.9%, and 46.2%, respectively. The MW distribution of the corresponding residual EOL samples obtained by GPC results confirmed that EOL was significantly depolymerized after treatment with Pep/hemin catalysts. As shown in Fig. 6c, the MW of untreated lignin expands from ~ 13,000 to ~ 600 Da with two peaks of MW at about 4000 and 1500 Da. H_2_O_2_ treatment alone did not change the MW distribution. It is, however, apparent that the MW peak at 4000 disappeared after Pep/hemin treatments. The **Pep-1/hemin** treatment, which led to the highest lignin mass loss, produced residue lignin with a lower peak MW at ~1400 Da confirming **Pep-1/hemin** indeed depolymerize the lignin. Both **Pep-1/hemin** and **Pep-2/hemin** treated lignin also show lower peak MW. The MW around 600 Da presented in both untreated and H_2_O_2_ control also disappeared in all Pep/hemin treated samples. A further analysis of the full width at half maximum (FWHM) of GPC peaks was used to evaluate the relative dispersity of depolymerized EOL. As shown in Supplementary Fig. 28, among these three catalysts, **Pep-1/hemin** showed the lowest FWHM, which is consistent with ABTS oxidation results to confirm that **Pep-1/hemin** showed the highest catalytic activity. The significant depolymerization of EOL induced by Pep/hemin catalysts was further confirmed by the analysis of GC-MS results of ethyl acetate extractable products. As shown in Fig. 6d, Pep/hemin catalysts depolymerized EOL into monomeric phenolic compounds, such as a–e. The disappearing of lower MW lignin, i.e. those with MW around 600 Da presented in both untreated and H_2_O_2_ treated samples in Fig. 6c, in Pep/hemin treated EOL samples was likely due to their depolymerization into these phenolic compounds. As a comparison, we treated EOL samples with LiP in the presence of H_2_O_2_ and performed GPC and GC-MS analyses. As shown in Supplementary Fig. 29a, LiP enzyme was not able to depolymerize EOL. GPC data confirmed that LiP-treated EOL sample showed similar results to those before treatment or only with H_2_O_2_. The GC-MS results (Supplementary Fig. 29b) showed that very little phenolic compounds were observed when EOL was treated with LiP in the presence of H_2_O_2_. These GPC and GC-MS results confirmed that LiP enzyme is not able to depolymerize lignin as Pep/hemin catalysts do.

To further confirm the depolymerization of EOL into phenolic compounds^62,63^, the yield of phenolic compounds following the depolymerization was quantified via microtiter-plated Folin-Ciocalteu (F-C) assay based on previously reported methods^64–66^ (see “Methods” for experimental details). The yields of phenolic compounds following the depolymerization of EOL by **Pep-1/hemin**, **Pep-2/hemin**, and **Pep-3/hemin** in the presence of H_2_O_2_ were calculated at 61.7%, 60.4% and 47.2%, respectively (Supplementary Table 1). The ability of Pep/hemin catalysts to depolymerize nearly two-thirds of a realistic lignin sample is extraordinary. However, for the sample treated with only H_2_O_2_, the yield was calculated at only 10.2% (Supplementary Table 1), indicating that the majority of EOL samples treated only with H_2_O_2_ are not the phenolic compounds as found in the Pep/hemin treated EOL samples^67–69^. These results confirmed that Pep/hemin can significantly depolymerize lignin in the presence of H_2_O_2_ which is a co-factor of most peroxidase. Given only 10.2% phenolic compounds were observed in the H_2_O_2_-treated EOL sample exhibiting a nearly 53.8% mass loss (Supplementary Table 1), we believe that the majority of EOL mass loss after only H_2_O_2_ treatment is likely due to the partial solubilization of lignin in H_2_O_2_^70–72^, instead of depolymerization as observed for EOL samples treated with Pep/hemin catalysts (Fig. 6c, d).

As far as we know, this is the only example showing the use of highly stable and tunable biomimetic enzymes for direct lignin depolymerization^73,74^. Although enzymatic methods have been used for depolymerizing lignin via white-rot fungi or bacteria^75,76^, this process often takes a long time (weeks) or is of low yield (from 7% to ~30%)^77,78^. On the other hand, chemical catalysis methods such as transition metals and acid/base are carried out under high pressure or at high temperatures^79^. Our results reveal that peptoid-based nanomaterial can depolymerize lignin to generate low-molecular-weight products at mild conditions and holds a promising potential for depolymerization of lignin to generate high-value products such as jet fuels. Figure 6e illustrates the possible mechanism of Pep/hemin that are contributing to its superior ability to depolymerize lignin polymer. First of all, the density of active sites on peptoid nanomaterial is higher than that on an enzyme due to the significant difference in MW between peptoid (~2 kDa) and ligninolytic enzyme such as lignin peroxidase (>38 kDa)^80^. A 10-nm-long Pep/hemin nanotube can carry ~910 reactive sites^16^ (one per 2 kDa) while a peroxidase molecule with a size of ~50 × 50 × 40 Å typically has one active site (0.05 per 2 kDa)^81^. In addition to higher density, the active sites of Pep/hemin are exposed on the nanomaterial surface, making them much more accessible compared to those of peroxidase enzymes which are encased inside the enzyme protein structures (Fig. 6a). The Pep/hemin complexes are likely smaller than a lignin polymer in size, thus they can easily attack multiple phenylpropane linkages. A possible interaction between the hemin active site and lignin subunit and subsequent cleavage of *α*C-*β*C linkage is shown in Fig. 6e. The ability of Pep/hemin complexes to conduct multiple bond cleavage in lignin is probably one major reason leading to the efficient lignin depolymerization. Finally, the electron transfer in the catalytic cycle can be hindered by the long-range electron transfer between lignin peroxidase and hemin active site^82^. In summary, our results demonstrated that Pep/hemin crystalline nanomaterials can effectively catalyze the oxidative depolymerization of real lignin and address some deficiencies of ligninolytic enzymes. Encouraged by these results and based on the tunability of peptoid nanomaterials, we expect to further enhance the catalytic activity of these peptoid-based enzyme mimetics by incorporating multiple coordinating ligands, such as Npyr and Nhis, in a single peptoid chain to further mimic the microenvironments of natural peroxidase enzymes.

## Discussion

By varying the structural features of peptoids including terminal ligands, side chain chemistries, and self-assembly behavior, we successfully develop a class of peptoid-based crystalline nanomaterials as peroxidase mimetics with tunable active sites and microenvironments. These biomimetic catalysts are highly stable and exhibit high efficiency in ABTS oxidation and lignin depolymerization. Catalytic oxidation results showed that the chain length of self-assembling peptoids played a key role in achieving high efficiency in the peroxidase-like activity, that is, the tubular Pep/hemin complexes assembled from peptoids with six Nce groups show the highest efficiency. Deviation from the optimal number of Nce groups reduces the catalytic activity of the formed nanotubes. We further demonstrated that self-assembled nanotubes containing Npyr terminal ligands exhibited the highest efficiency in both ABTS oxidation and lignin depolymerization while changing Npyr to Nhis or Ntrp led to reduced activity. Our results also revealed that these peptoid-based peroxidase mimetics are highly stable and remain active at elevated temperatures and various solution pHs (from 3.0 to 10.0). Kinetic studies showed that these enzyme mimetics exhibited a ping-pong (double displacement) mechanism in catalytic oxidation. Because peptoid-based crystalline nanomaterials are highly tailorable and stable, we expect the self-assembly of peptoids into hierarchically structured crystalline nanomaterials with the ordered alignment and organization catalytic sites will provide a fascinating strategy for the design and synthesis of robust enzyme mimetics for various applications including lignin depolymerization.

## Methods

### Materials

Hemin and dimethyl sulfoxide were purchased from Sigma Chemical Co. (St. Louis, MO). *β*-Alanine *t*-butyl ester hydrochloride was purchased from Chem-Impex International, Inc. This hydrochloride salt was deprotected by addition of sodium hydroxide aqueous solution, then extracted with CH_2_Cl_2_, filtered, and rotary evaporated for further use. *N*,*N*ʹ-diisopropylcarbodiimide, bromoacetic acid, and trifluoroacetic acid (TFA) were purchased from Chem-Impex International, Inc and used as received. 4-Bromobenzylamine was purchased from Oakwood Products, Inc. All other amine submonomers and other reagents are obtained from commercial sources and used without further purification. MilliQ water at 18.2 MΩ cm was used for all experiments.

### Peptoids sequence synthesis and self-assembly

All peptoids were synthesized using a modified solid-phase sub-monomer synthesis method as described previously^16^. Peptoid syntheses were performed either on a Aapptec Apex 396 robotic synthesizer or manually in 6-ml plastic vials. After solid-phase synthesis, all peptoids were cleaved from resins by mixing with 95% TFA in water for over 30 min. After evaporating TFA, the obtained peptoid crudes were dissolved in water and acetonitrile (1:1, v/v) for HPLC purification. All peptoids were purified using reverse-phase HPLC on an XBridge™ Prep C18 OBD™ (10 μm, 19 mm × 100 mm), having a narrow gradient of acetonitrile in H_2_O with 0.1% TFA over 20 min with a 20 ml min^−1^ flow rate. Waters ACQUITY reverse-phase UPLC (using a corresponding gradient at 0.4 ml min^−1^ over 7 min with an ACQUITYBEH C18, 1.7 μm, 2.1 mm × 50 mm column) connected with a Waters SQD2 mass spectrometry system was used to analyze the purified peptoids. The final peptoid product was lyophilized at least twice from the mixture of H_2_O/CH_3_CN solution. The obtained lyophilized sample was finally divided into small portions (1.0 or 2.0 μmol) and stored at −80 °C.

Lyophilized and HPLC-grade peptoids were dissolved in the mixture of H_2_O/CH_3_CN (1:1, v/v) to make a 5.0 mM clear solution, which was then transferred to a 4 °C refrigerator for slow evaporation. Suspensions or gel-like materials containing crystalline nanotubes or nanosheets were formed after 3 days. For hemin-peptoid co-assembly, lyophilized peptoid and equimolar hemin were dissolved in H_2_O/CH_3_CN (1:1, v/v), and the mixture was left at 4 °C following the same protocol described above for slow evaporation to obtain brown and gel-like materials. For co-assembly of peptoids and metal ions, the equimolar CuSO_4_ or MnCl_2_ aqueous solution was mixed with peptoid and left at 4 °C for slow evaporation to obtain gel-like materials.

### Preparation of Pep-1 + hemin

Pre-formed **Pep-1** nanotubes were incubated with equimolar hemins in a 500 µl aqueous solution. After about 6-h incubation, this solution was centrifuged and washed well with water (500 µl) three times. The obtained hemin-containing nanotubes (namely **Pep-1** **+** **hemin**) were then used for catalysis measurements.

### Pep/hemin catalyst stability test

The corresponding gel-like Pep/hemin materials (1 µmol) were diluted with 500 µl H_2_O to yield a 0.2 mM solution. Then the obtained aqueous solution was incubated at 90 °C for 2 h, or at room temperature for 30 days, or heated at 60 or 90 °C for overnight before being used for catalytic studies or TEM imaging.

### Transmission electron microscopy (TEM)

For TEM characterizations, TEM samples were made by having a 2.0 μl drop of the self-assembled sample diluted with 5.0 μl of deionized water. Once this solution was deposited onto carbon-coated copper grids for 10 min, it was dried with filter paper. Afterward, 5.0 μl of 2% phosphotungstic acid (PTA) in distilled water (pH 7.0) was dropped onto the TEM grid for another 2 min for negative staining. After removing the extra PTA solution using filter paper, the TEM grid was dried and ready for TEM imaging. All TEM data were collected on a 200-kV FEI Tecnai TEM microscope.

### Atomic force microscopy (AFM)

AFM images were collected on a Bruker MultiMode 8 by using either a ScanAsyst mode or a tapping mode. For AFM sample preparation, a 1.0 μl drop of the self-assembled peptoid solution was diluted with 40 μl H_2_O. Then it was deposited onto a freshly cleaved mica surface and incubated for 10 min. After removing the solution using filter paper, the mica sample was dried using a stream of N_2_ gas and ready for AFM imaging.

### Scanning electron microscopy (SEM)

Water-washed and diluted peptoid nanotubes or nanosheets were drop-cast onto silicon substrates to obtain SEM samples. The morphology of all samples was examined by using an FEI Helios NanoLab 600i SEM. The chemical composition of all samples was analyzed by using EDS with an Oxford X-Max 80 EDS detector (Oxford Instruments Analytical Ltd).

### X-ray powder diffraction (XRD)

All XRD data were collected on a multiple wavelength anomalous diffraction and monochromatic macromolecular crystallography beamline, 8.3.1 (possessing a 5 T single pole superbend source with an energy range of 5–17 keV) at the Advanced Light Source located at Lawrence Berkeley National Laboratory. All XRD data were collected with a 3 × 3 CCD array (ADSC Q315r) detector at a wavelength of 1.1159 Å. For XRD sample preparations, samples of peptoid assemblies were loaded onto a Kapton mesh (MiTeGen) and dried for XRD measurements. All XRD Data were analyzed using custom Python scripts.

### UV-Visible (UV-Vis) Spectroscopy

UV-Vis spectroscopy was either performed on a TECAN Safire2 spectrometer using a 96-well plate or an Ultrospec 2100 pro UV/Visible spectrophotometer. For TECAN spectrometer with a plate reader, to each well of the plate, 100 μl of the solution was pipetted, and a full spectrum from 200 nm to 800 nm was recorded with triplicate samples. For Ultrospec 2100 spectrometer, a quartz cuvette with a path length of 1.0 cm was used for all measurements.

### Specific activity (initial velocity screening)

The specific activity (µmol mg^−1^ min^−1^) was calculated according to the equation:

4 $${a}_{{{{{{{\rm{catalyst}}}}}}}}=\frac{{b}_{{{{{{{\rm{catalyst}}}}}}}}}{\left[m\right]}$$

where *a*_catalyst_ is the activity expressed in units per milligram (U mg^−1^), and [*m*] is the weight of catalyst used in each assay (mg). The *b*_catalyst_ is determined as follows:

5 $${b}_{{{{{{{\rm{catalyst}}}}}}}}=\,\frac{\triangle A/\triangle t}{\varepsilon \times l}\times V$$

where *b*_catalyst_ is the catalytic activity of peptoids expressed in unites; *A* is the absorbance after subtraction of the blank value; Δ*A*/Δ*t* is the initial rate of change in the absorbance at 420 nm for ABTS and 652 nm for TMB expressed in min^−1^; *ε* is the molar absorption coefficient of the substrate (36,000 M^−1^ cm^−1^ at 420 nm for ABTS, and 39,000 M^−1^ cm^−1^ at 652 nm for TMB).

In a typical experiment, the gel-like peptoid/hemin material (1 µmol) was diluted to 500 µl using H_2_O to yield a 0.2 mM solution. Then, 1000 µl 0.2 mM ABTS solution is mixed with 10 µl 30% H_2_O_2_ in a quartz cell with a path length of 1 cm. A single shot of 100 µl 0.2 mM peptoid/hemin catalyst was injected into the ABTS/H_2_O_2_ substrate mixture to initiate the reaction, at which time the absorbance was recorded every 5 s at 420 nm and 652 nm for ABTS and TMB, respectively. To determine the reaction rate of Pep/hemin under different temperatures (i.e. 60, and 90 °C), 0.2 mM **Pep-1/hemin** catalysts were incubated at 60 °C or 90 °C for 2 h. Immediately after the incubation, **Pep-1/hemin** samples were added to preheated ABTS solutions (60 °C and 90 °C respectively) to determine the initial reaction velocity.

### Lignin depolymerization and characterizations

An organosolv lignin (lodgepole pine) was collected during pretreatment of lodgepole pine by 65% ethanol and 1.1% sulfuric acid (on wood) at 170 °C for 60 min^61^. The purified and freeze-dried EOL sample was provided by Prof. Xuejun Pan from University of Wisconsin-Madison and used for depolymerization experiments in this study. Lignin peroxidase (>0.1 U mg^−1^) was purchased from Creative Biomart Inc (Shirley, NY, United States) and stored at −20 °C until use. In a typical lignin depolymerization experiment, 50 mg of the EOL was mixed with 1000 µl 0.1 mM Pep/hemin or 2 mg ml^−1^ LiP solution in a glass vial and incubated at 200 rpm for 30 min at 60 °C. Thereafter, 50 µl of 30% H_2_O_2_ was added to the reaction mixture and the vial was further incubated at 200 rpm for 30 min, 1 h, or 2 h at 60 °C.

The depolymerization products of lignin were extracted with 1 ml ethyl acetate (3x), and the organic layer was carefully combined and dried under N_2_ flow, followed by silylation with 100 µl of bis(trimethylsilyl) trifluoroacetamide + 1% trimethylchlorosilane (BSTFA + 1% TMCS, Regis). The remaining aqueous layer and lignin residue were washed with 1 ml ethyl acetate (2x) and 1 ml water (2x), lyophilized, and weighed.

F-C experiments were performed to determine the yield of phenolic products from lignin depolymerization following established methods^64–66^. In a typical F-C experiment, residual lignin sample was washed with water for two times and the aqueous phase was combined yielding a total volume of 10 ml. Afterward, 20 μl of the aqueous phase was added to 100 μl F-C reagent, 1.5 ml water and 300 μl Na_2_CO_3_ (20%). The mixture is incubated at 45 °C for 1 h. After this, 100 μl of the mixture was pipetted to a 96-well plate and the absorbance at 765 nm was measured against a blank water sample. The amounts of phenolic compounds were quantified by correlating the sample absorbance with phenolic compounds concentration using a standard curve generated from isoeugenol (a standard compound) at different concentrations.

### Gas chromatography-mass spectroscopy (GC-MS)

GC-MS was performed on an Agilent 7890A/5975C spectrometer using a DB-5 capillary column and it was used to analyze silylated ethyl acetate fraction (see Lignin depolymerization and characterizations section above). GC oven temperature setting was as follows: 100 °C for 3 min, 5 °C min^−1^ ramp to 200 °C, 200 °C for 3 min, 20 °C min^−1^ ramp to 320 °C, and 320 °C for 10 min. Solvent delay was set to 3 min. Positive EI mode was selected for mass spectrometer detector with temperature setting as follows: MS Source Setpoint was set at 230 °C and MS Quad Setpoint was set at 150 °C. Scanning ion range was set from *m*/*z* 50–1000. Compounds between 5 and 30 min were identified and chromatograms were plotted.

### Gel permeation chromatography (GPC)

The analysis was performed according to an established method^83^ using a Perkin Elmer HPLC system with a PDA LC detector. Three Agilent columns (60, 300, and 1000 Å porosity; 5 μm bead size) were used to perform the size exclusive separation at room temperature. The mobile phase was tetrahydrofuran (THF), applied at a flow rate of 0.6 ml min^−1^. Samples were prepared at a concentration of 30 mg ml^−1^ in THF. A 100 µl injection volume was used. The data was acquired and analyzed using the TotalChrom Navigator software package.

All the lignin samples were acetylated prior to the analysis. To prepare an acetylated lignin sample, the residual lignin sample was mixed with 1 ml of pyridine and 1 ml of acetic anhydride and stirred for 24 h. After acetylation, 5 ml MeOH was added to the mixture, and the solvent was removed under reduced pressure. The remaining solid was dissolved in 1 ml THF and was used for GPC analysis. Standard samples of polystyrene beads (Fischer Scientific) with known *M*_w_ of 1300, 3350, 5200, 13,000, and 50,000 and veratrole with *M*_w_ of 138 were also dissolved in THF at 30 mg ml^−1^ concentration for GPC analysis.

### Reporting summary

Further information on research design is available in the Nature Research Reporting Summary linked to this article.

## Supplementary information

Supplementary Information

Description of Additional Supplementary Information

Reporting Summary

## Data Availability

All data are available within the Article and Supplementary Files, or available from the corresponding authors on request.

## Code availability

Custom code is available from the corresponding authors on request.
