# Supplementary material for: Highly stable and tunable peptoid/hemin enzymatic mimetics with natural peroxidase-like activities
Source: Nat Commun. 2022 May 31;13:3025. doi: 10.1038/s41467-022-30285-9 (PMC9156750; doi:10.1038/s41467-022-30285-9)
Supplement: Supplementary file 1 — Supplementary Information [file 41467_2022_30285_MOESM1_ESM.docx]

Supplementary Information

Highly Stable and Tunable Peptoid/Hemin Enzymatic Mimetics with Natural Peroxidase-Like Activities

Tengyue Jian,^1^† Yicheng Zhou,^1,2,^† Peipei Wang,^2^† Wenchao Yang,^1,3^ Peng Mu,^1,4^ Xin Zhang^1^, Xiao Zhang^✉2^ and Chun-Long Chen^✉1^

^1^Physical Sciences Division, Pacific Northwest National Laboratory, Richland, WA 99352, United States.

^2^The Voiland School of Chemical Engineering and Bioengineering, Washington State University, Richland, WA 99354, United States.

^3^School of Chemical Engineering and Technology, State Key Laboratory of Chemical Engineering, Tianjin University, Tianjin 300072, People’s Republic of China.

^4^Department of Mechanical Engineering and Materials Science and Engineering Program, State University of New York, Binghamton, NY13902, United States.

^✉^email: [chunlong.chen@pnnl.gov](mailto:chunlong.chen@pnnl.gov); [x.zhang@wsu.edu](mailto:x.zhang@wsu.edu)

†These authors contributed equally to this work.

**List of supplementary materials:**

Supplementary Fig. 1. TEM images of Pep-1/hemin nanotube assembly.

Supplementary Fig. 2. UV-Vis spectra of the mixture of hemin and Pep-1 at different molar ratios.

Supplementary Fig. 3. AFM images of Pep-1/Cu, Pep-1/Mn, and Pep-1+hemin assemblies. Supplementary Fig. 4. XRD patterns of Pep-1/Cu, Pep-1/Mn, and Pep-1/hemin nanotubes.

Supplementary Fig. 5. SEM images and EDS measurements of assembled nanotube catalysts.

Supplementary Fig. 6 to Supplementary Fig. 15. UPLC-MS characterization of Pep-1 to Pep-10.

Supplementary Fig. 16 to Supplementary Fig. 23. AFM images of Pep/hemin nanotubes and their corresponding height profiles.

Supplementary Fig. 24. AFM images of Pep-10/hemin nanosheet and its height profile.

Supplementary Fig. 25. XRD data of Pep-10/hemin nanosheet.

Supplementary Fig. 26. TEM images of Pep/hemin nanotubes after ABTS oxidation reactions.

Supplementary Fig. 27. Correlation between the slope values obtained in the four Lineweaver-Burk plots against inversed H_2_O_2_ concentration changes associated with Fig. 4a.

Supplementary Fig. 28. Gel permeation chromatography (GPC) profile of ethanol organosolv lignin (EOL) samples after treatment with Pep-1/hemin, Pep-2/hemin, or Pep-3/hemin in the presence of H­_2_O_2_.

Supplementary Fig. 29. GPC profiles of ethanol organosolv lignin (EOL) samples before and after treatments, and GC-MS results of ethyl acetate extractable products from treated EOL samples.

Supplementary Table 1. Mass balance obtained during the depolymerization of lignin with different conditions.

**Supplementary Figures**


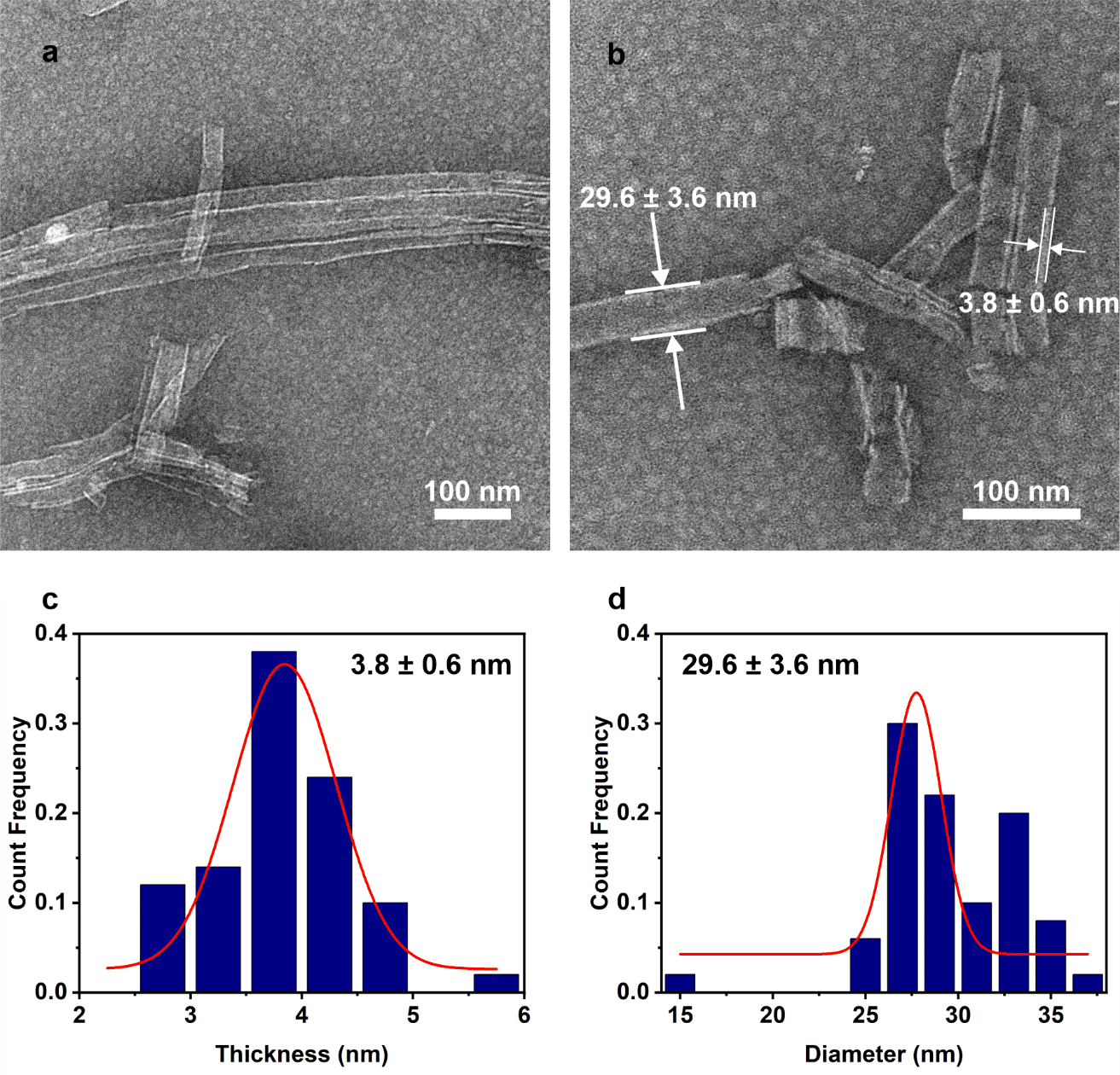


**Supplementary Fig. 1. TEM images of Pep-1/hemin nanotube assembly.** (a, b) TEM images showing the well-defined tubular structure of **Pep-1/hemin** co-assembly with tube diameter of 29.6 ± 3.6 nm and wall thickness of 3.8 ± 0.6 nm. (c) Wall thickness and (d) diameter distributions of the **Pep-1/hemin** nanotube based on 50 counts.

**b**

**a**

**Supplementary Fig. 2**. **UV-Vis spectra of the mixture of hemin and Pep-1 at different molar ratios.** (a) UV-vis spectra of **Pep-1** and hemin in H_2_O/CH_3_CN (v:v = 1:1) solution at different hemin:Pep-1 molar ratios (from 0 to 2.0), along with pure hemin in H_2_O/CH_3_CN (v:v = 1:1) solution. (b) Absorbance at 503 nm as a function of hemin:Pep-1 molar ratio.


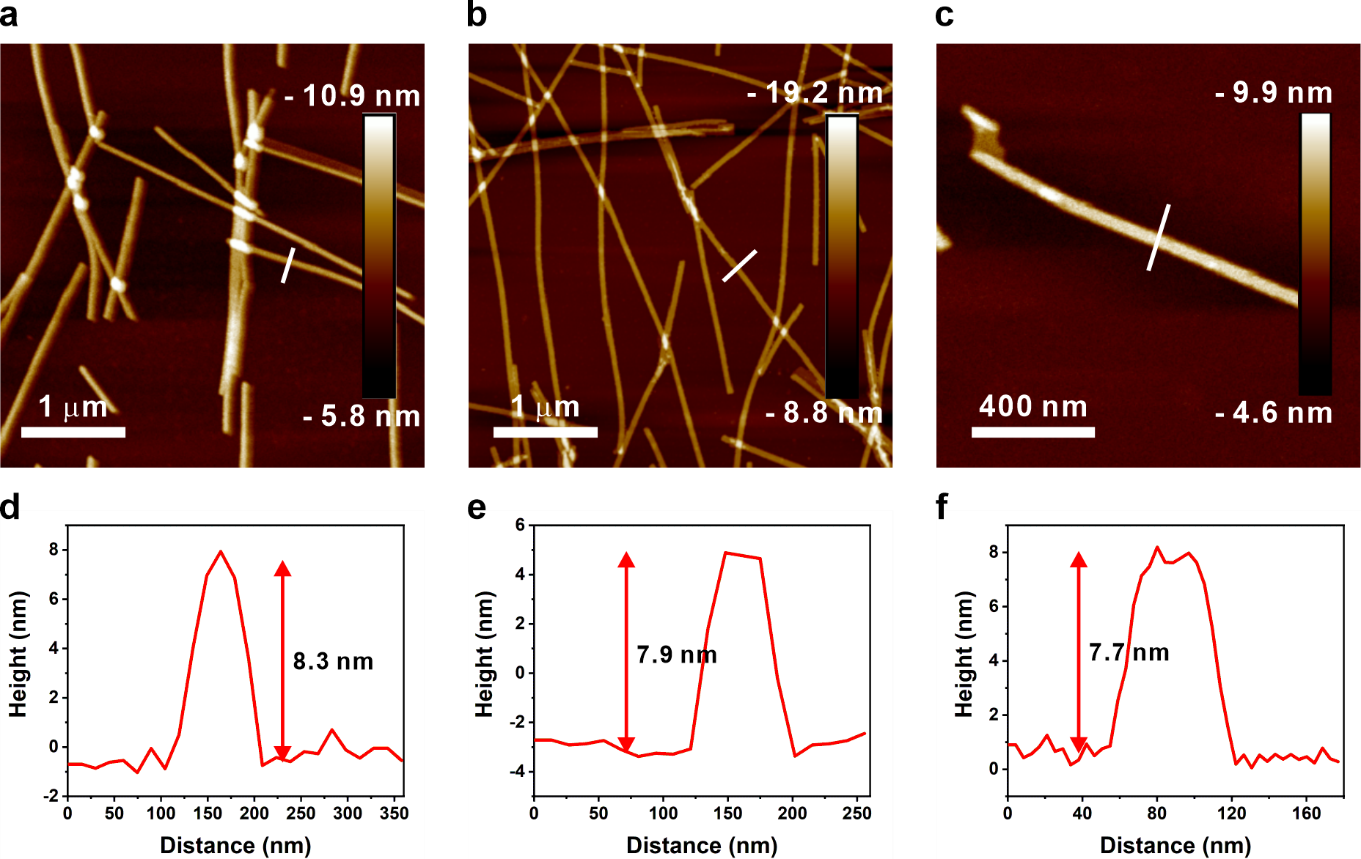


**Supplementary Fig. 3.** **AFM images of (a) Pep-1/Cu, (b) Pep-1/Mn, and (c) Pep-1+hemin assemblies.** **Pep-1/Cu** and **Pep-1/Mn** indicate equimolar assembly of **Pep-1** with Cu^2+^ and Mn^2+^ respectively. And **Pep-1+hemin** indicates simple mixing of equimolar **Pep-1** and hemin. Height profiles corresponding to line scans in (a), (b), and (c) are plotted in (d), (e), and (f), respectively.

**Supplementary Fig. 4.** X-ray diffraction (XRD) patterns of **Pep-1/Cu**, **Pep-1/Mn**, and **Pep-1/hemin** nanotubes.


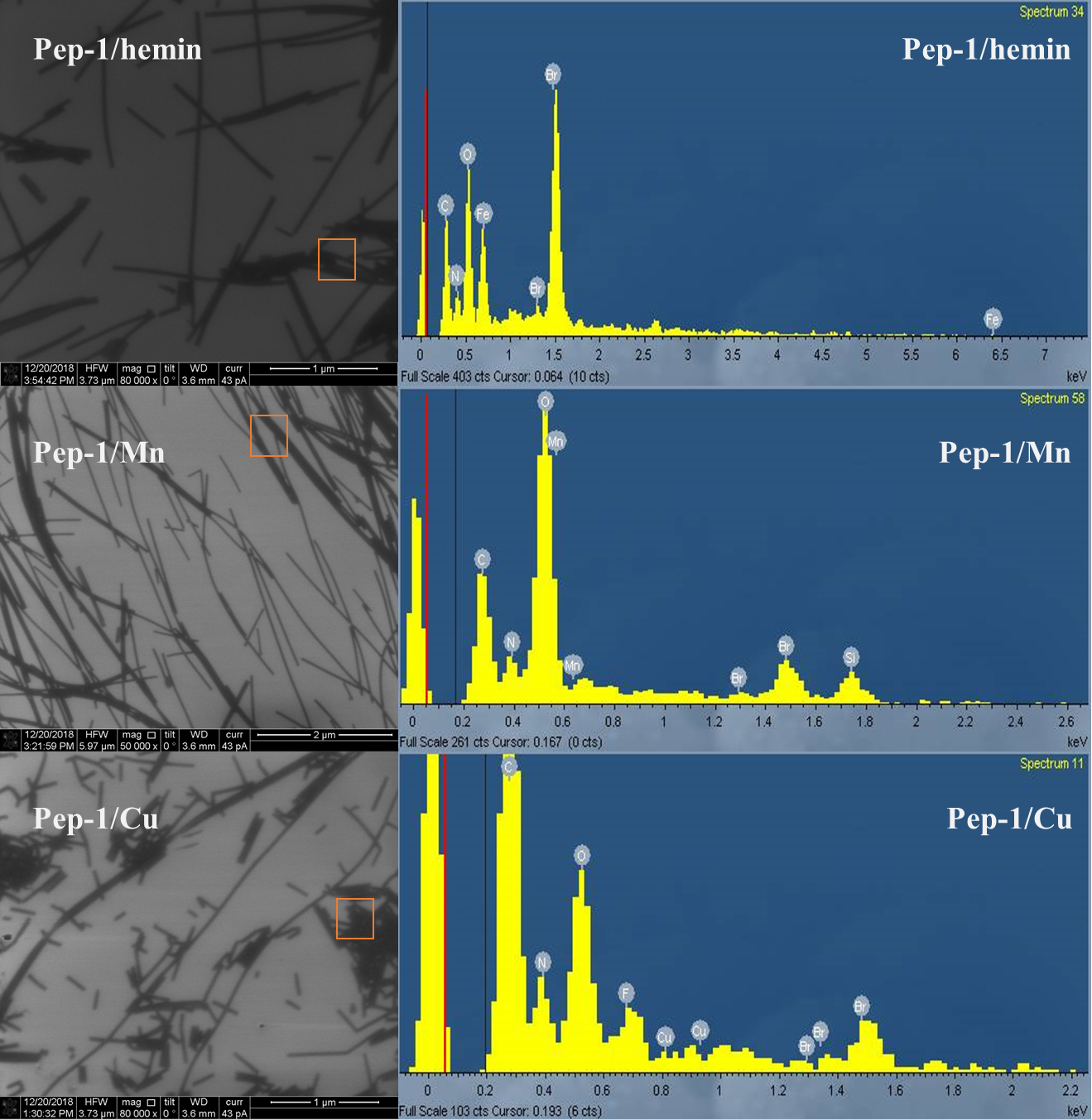


**Supplementary Fig. 5**. Scanning electron microscopy (SEM) images (left) and energy-dispersive X-ray spectroscopy (EDS) measurements (right) in the designated boxed regions in SEM images of assembled and water-washed **Pep-1/hemin** (top), **Pep-1/Mn** (middle), and **Pep-1/Cu** (bottom) nanotube catalysts.


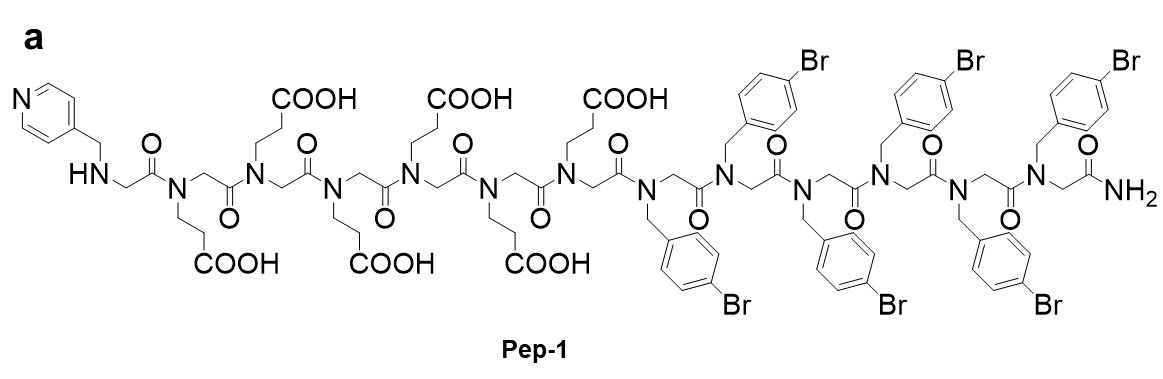


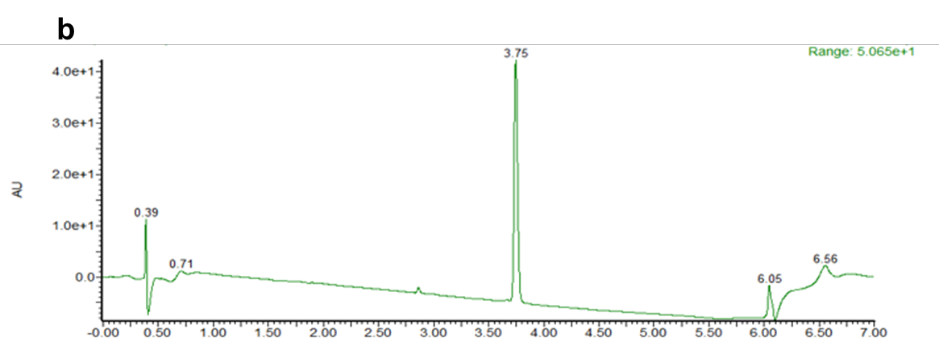


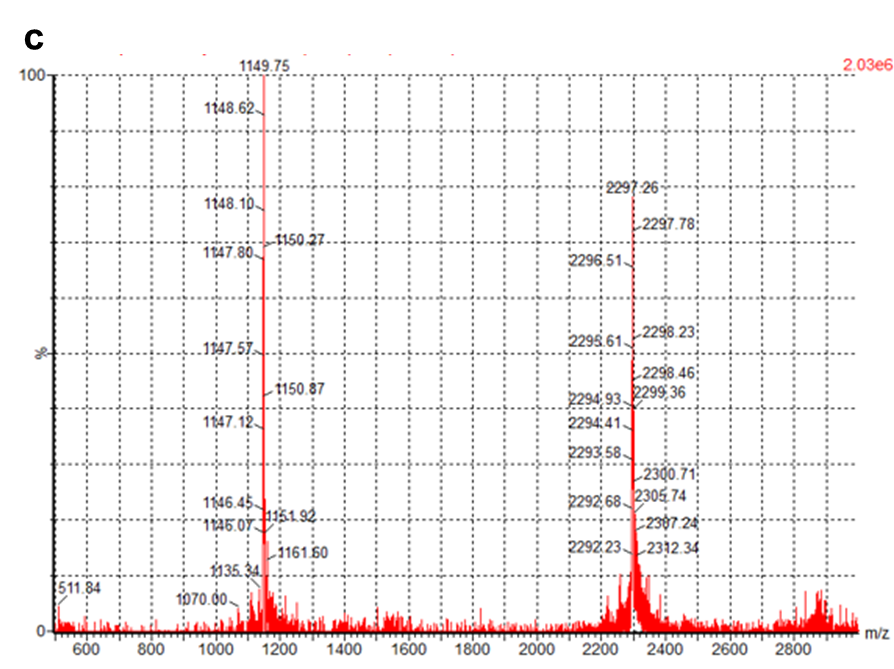


**Supplementary Fig. 6.** **UPLC-MS characterization of Pep-1.** (a) Structure of **Pep-1**. (b) UPLC characterization of **Pep-1** with the gradient of 5 - 95% CH_3_CN in H_2_O. (c) MS characterization of **Pep-1**. 2296.32 (Molecular Weight), 2297.26 (Found: [M+H]^+^).


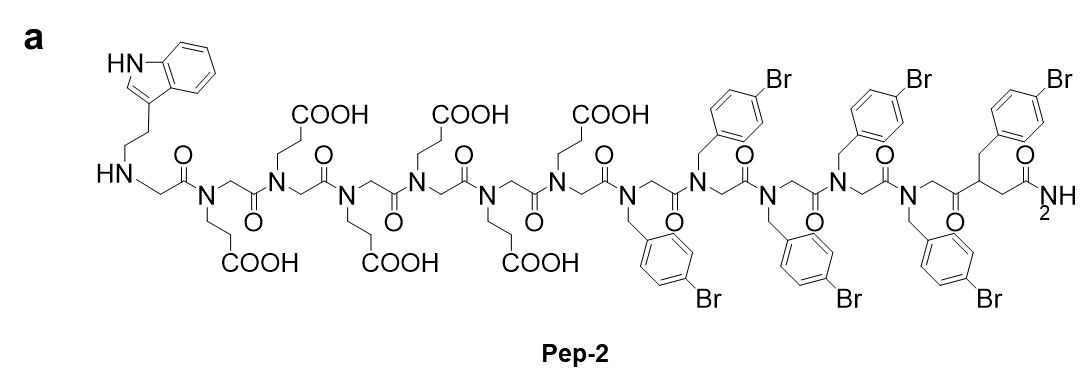


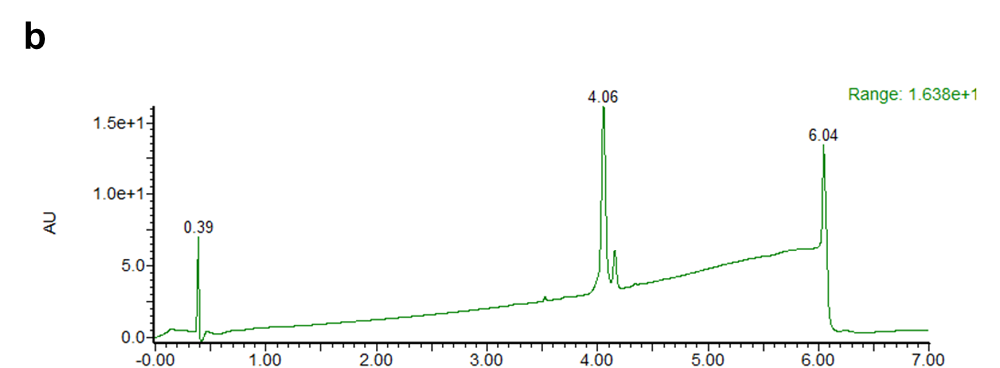


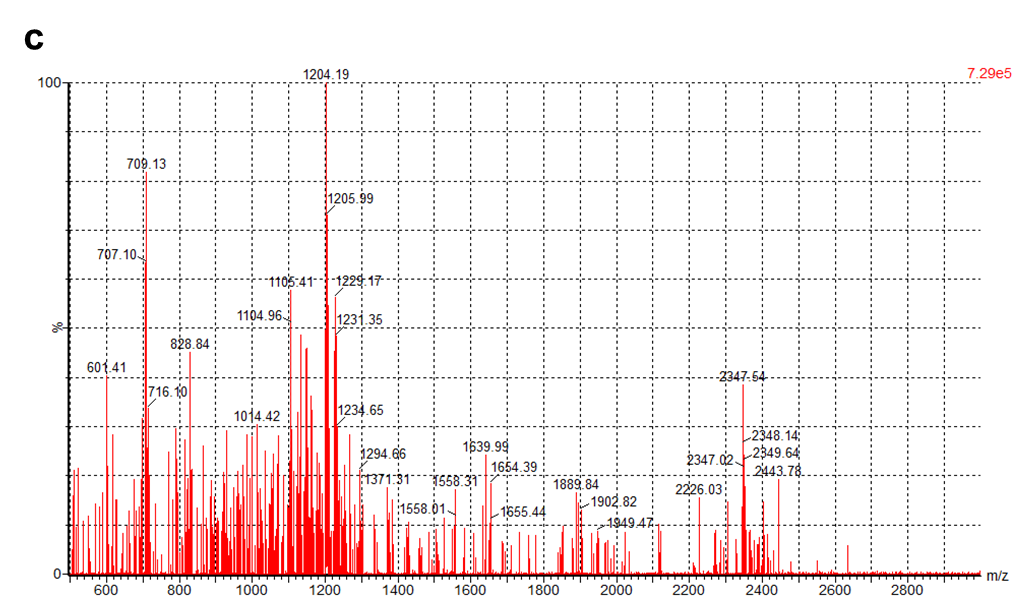


**Supplementary Fig. 7. UPLC-MS characterization of Pep-2.** (a) Structure of **Pep-2**. (b) UPLC characterization of **Pep-2** with the gradient of 5 - 95% CH_3_CN in H_2_O. (c) MS characterization of **Pep-2**. 2347.41 (Molecular Weight), 2347.54 (Found: [M]^+^).


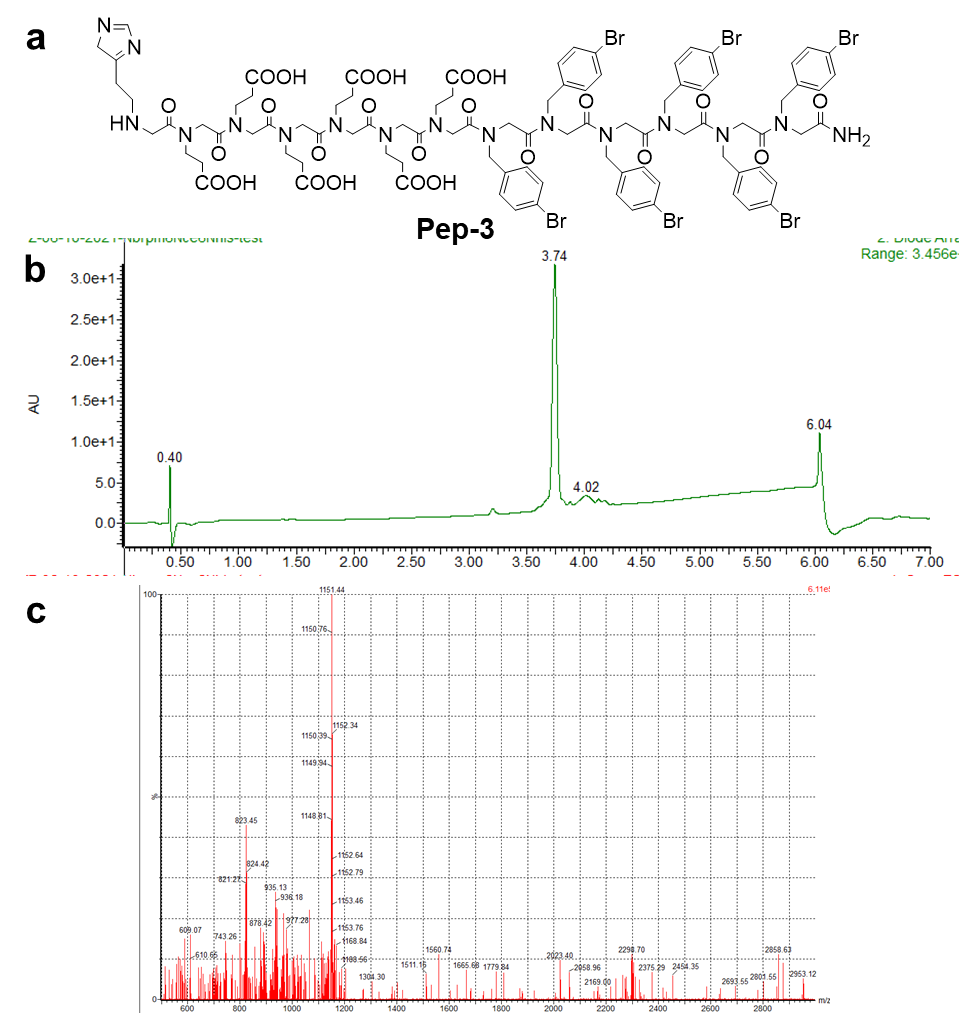


**Supplementary Fig. 8. UPLC-MS characterization of Pep-3.** (a) Structure of **Pep-3**. (b) UPLC characterization of **Pep-3** with the gradient of 5 - 95% CH_3_CN in H_2_O. (c) MS characterization of **Pep-3**. 2298.34 (Molecular Weight), 2298.79 (Found: [M]^+^).


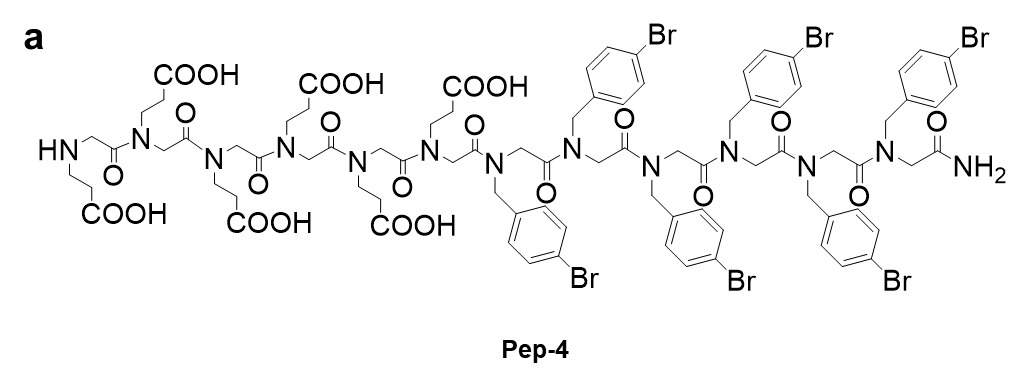


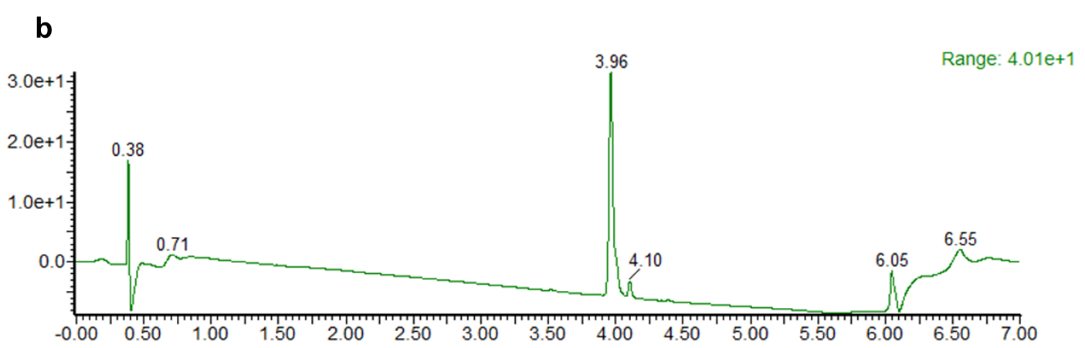


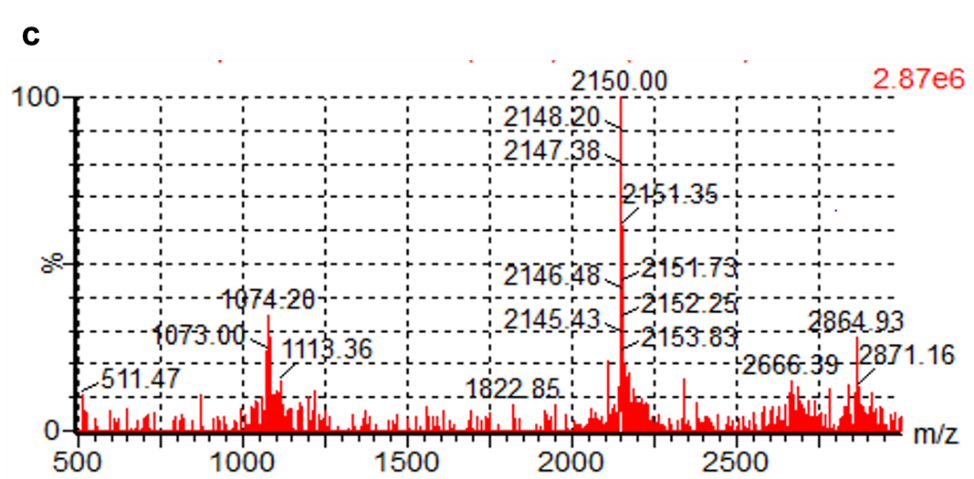


**Supplementary Fig. 9. UPLC-MS characterization of Pep-4.** (a) Structure of **Pep-4**. (b) UPLC characterization of **Pep-4** with the gradient of 5 - 95% CH_3_CN in H_2_O. (c) MS characterization of **Pep-4**. 2148.16 (Molecular Weight), 2148.20 (Found: [M]^+^).


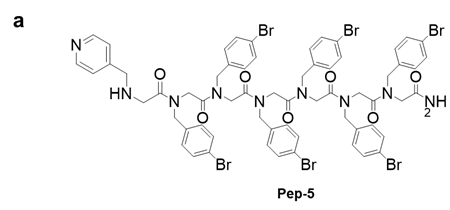


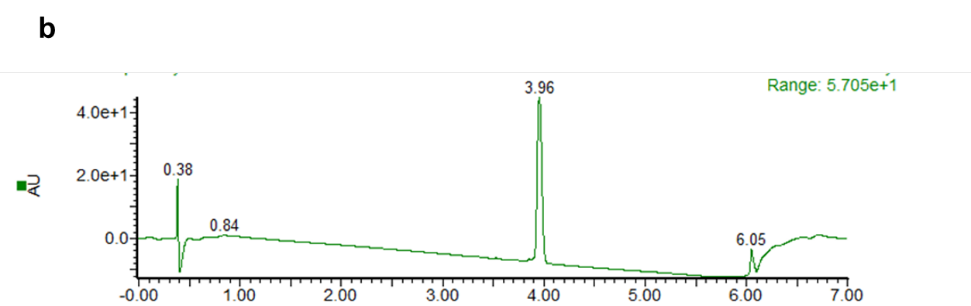


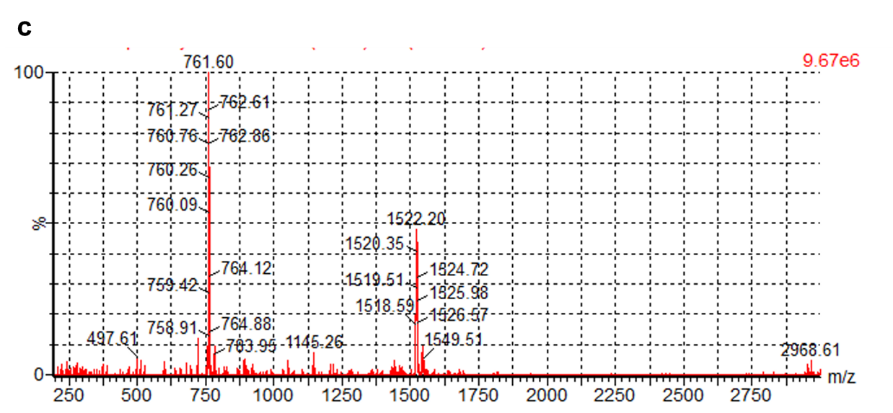


**Supplementary Fig. 10. UPLC-MS characterization of Pep-5.** (a) Structure of **Pep-5**. (b) UPLC characterization of **Pep-5** with the gradient of 5 - 95% CH_3_CN in H_2_O. (c) MS characterization of **Pep-5**. 1521.63 (Molecular Weight), 1522.20 (Found: [M+H]^+^).


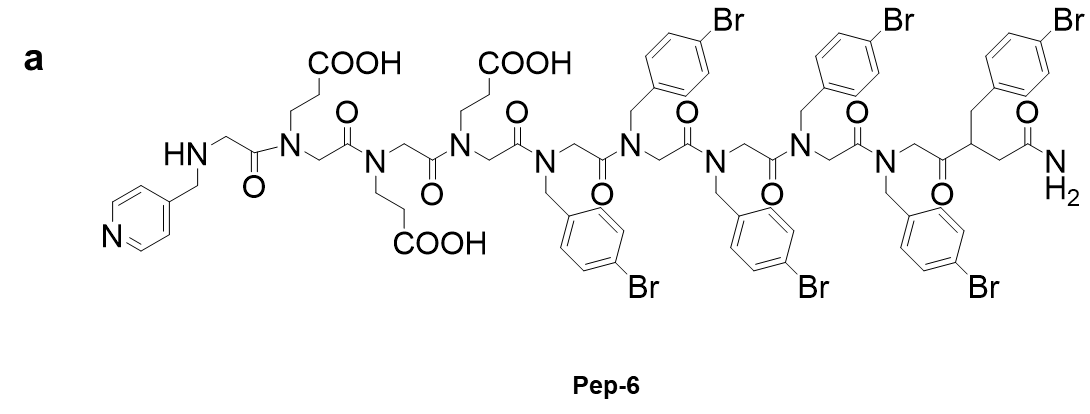


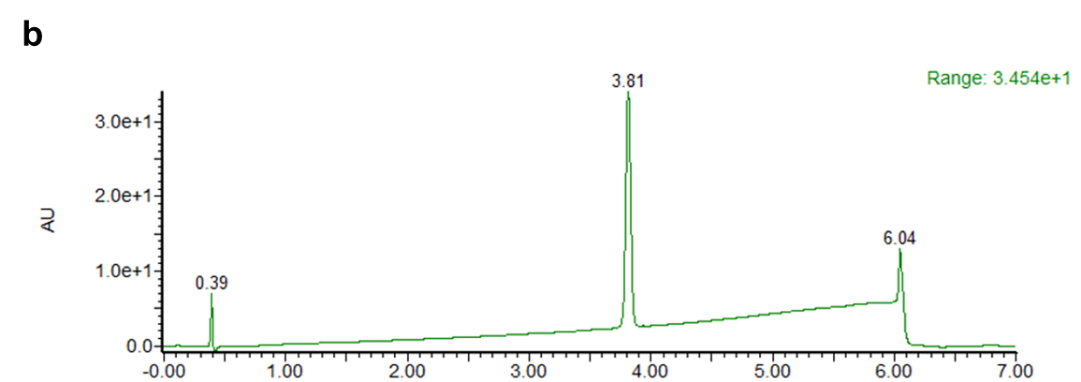


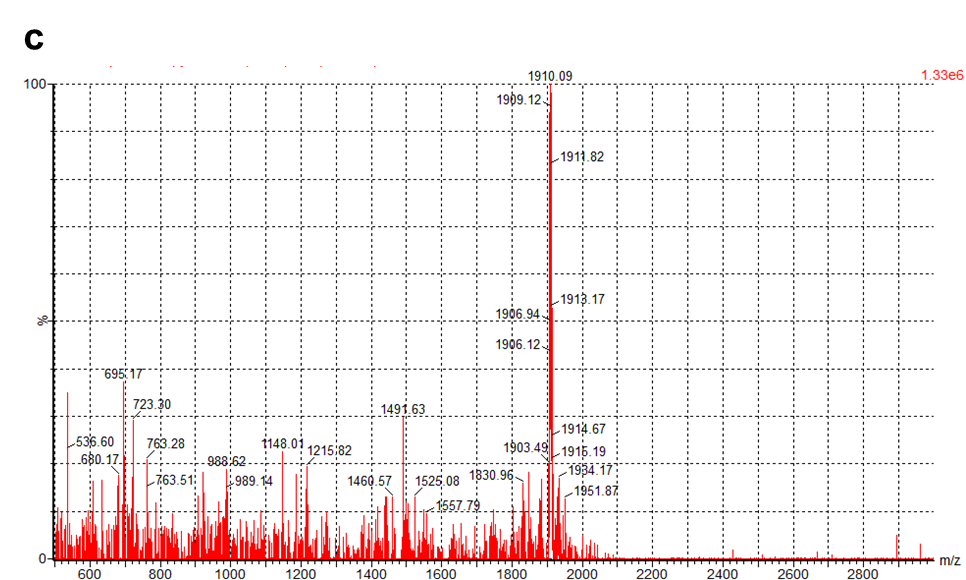


**Supplementary Fig. 11. UPLC-MS characterization of Pep-6.** (a) Structure of **Pep-6**. (b) UPLC characterization of **Pep-6** with the gradient of 5 - 95% CH_3_CN in H_2_O. (c) MS characterization of **Pep-6**. 1908.01 (Molecular Weight), 1909.12 (Found: [M+H]^+^).


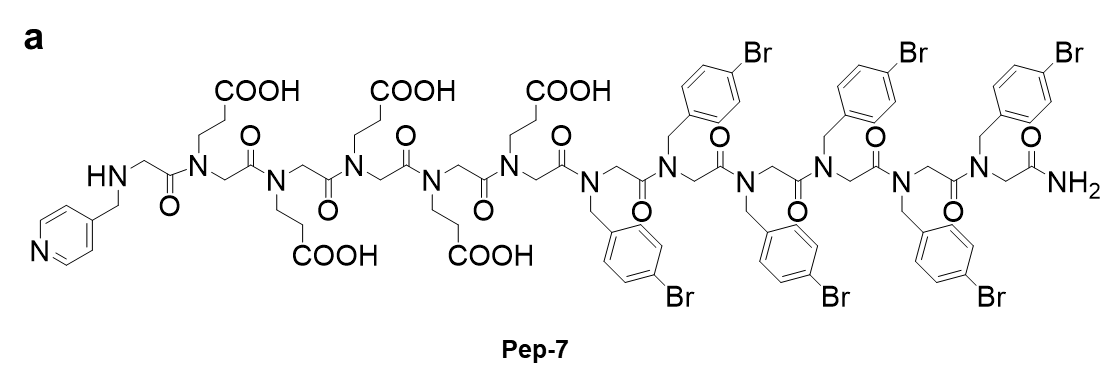


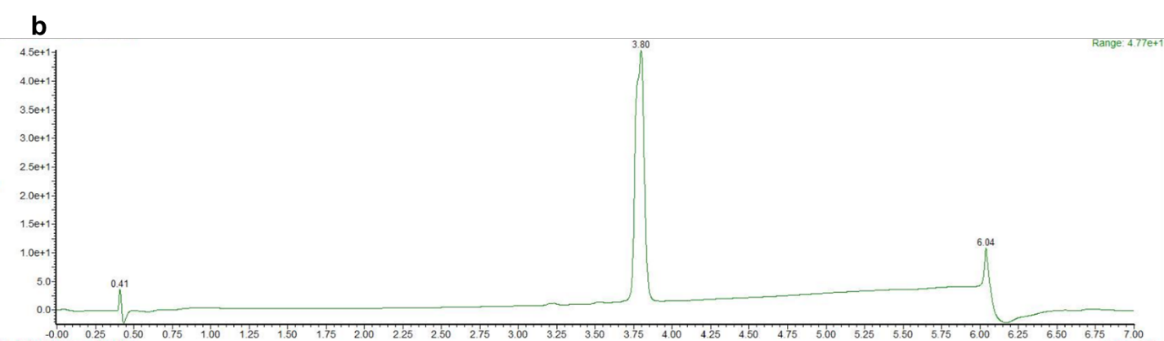


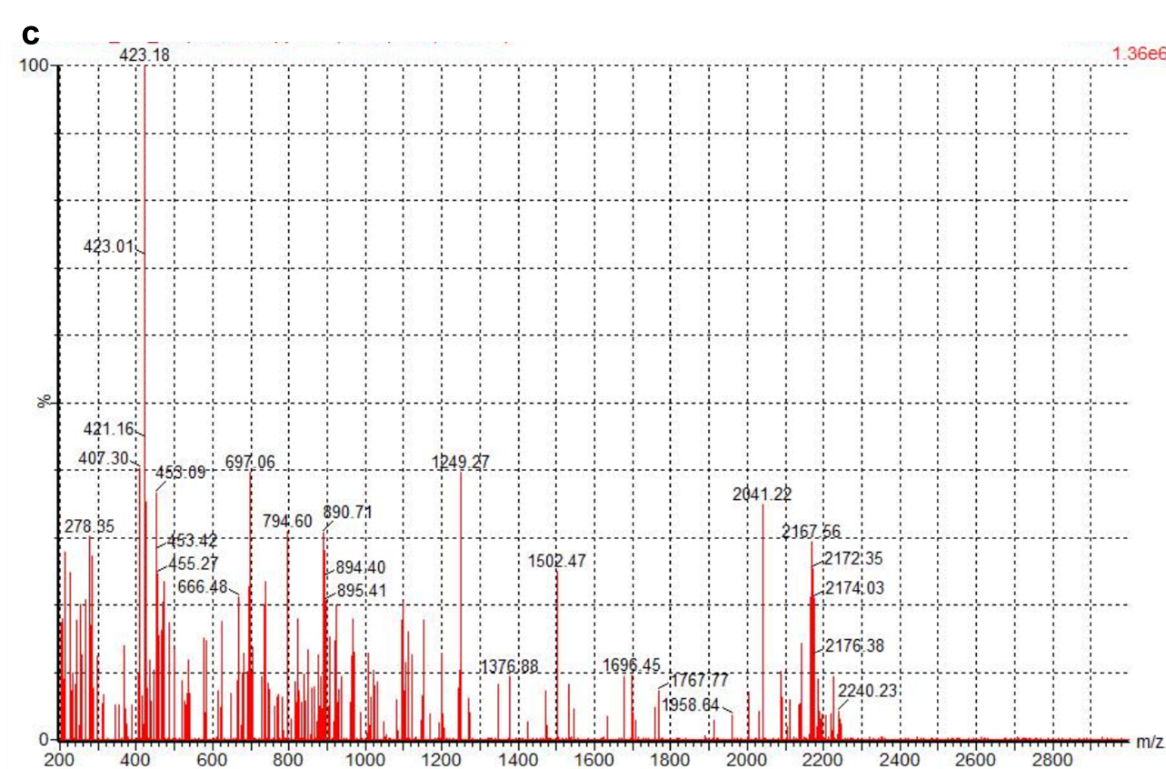


**Supplementary Fig. 12. UPLC-MS characterization of Pep-7.** (a) Structure of **Pep-7**. (b) UPLC characterization of **Pep-7** with the gradient of 5 - 95% CH_3_CN in H_2_O. (c) MS characterization of **Pep-7**. 2166.22 (Molecular Weight), 2167.66 (Found: [M+H]^+^).


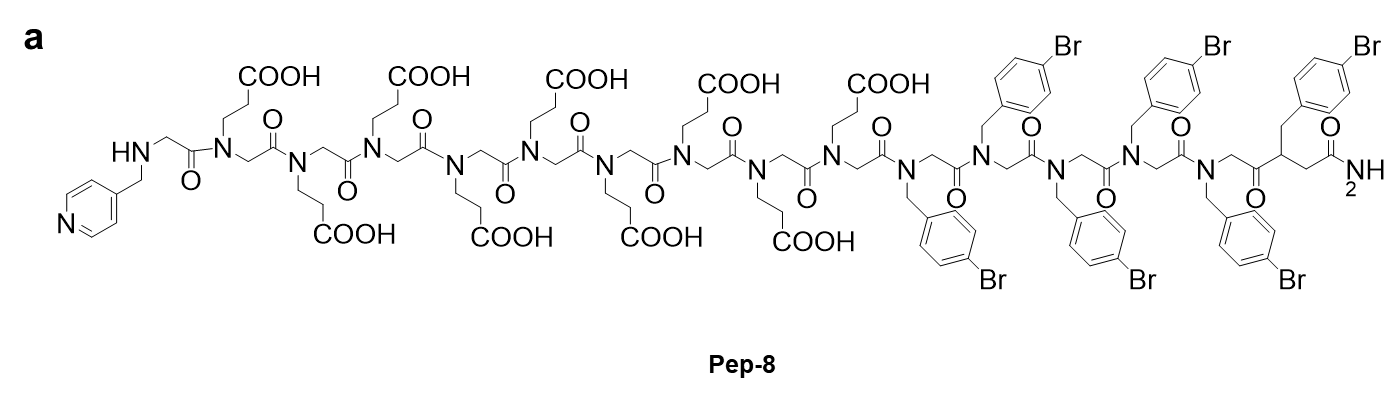


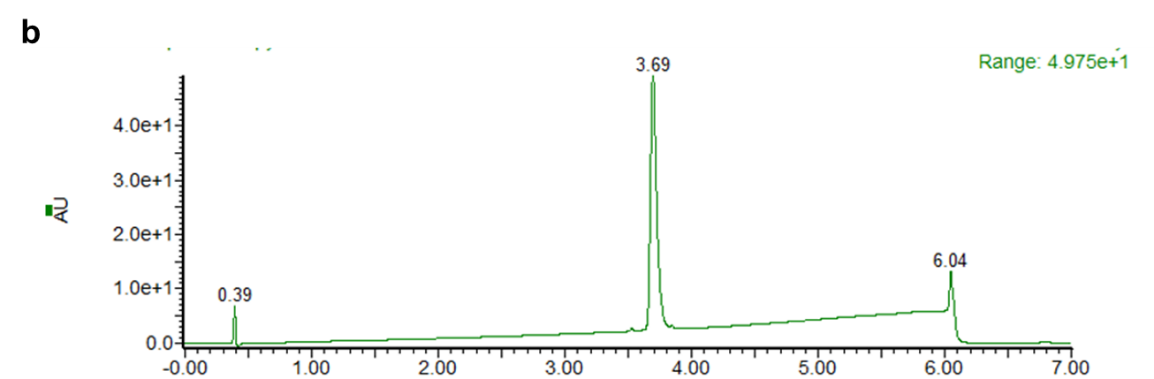

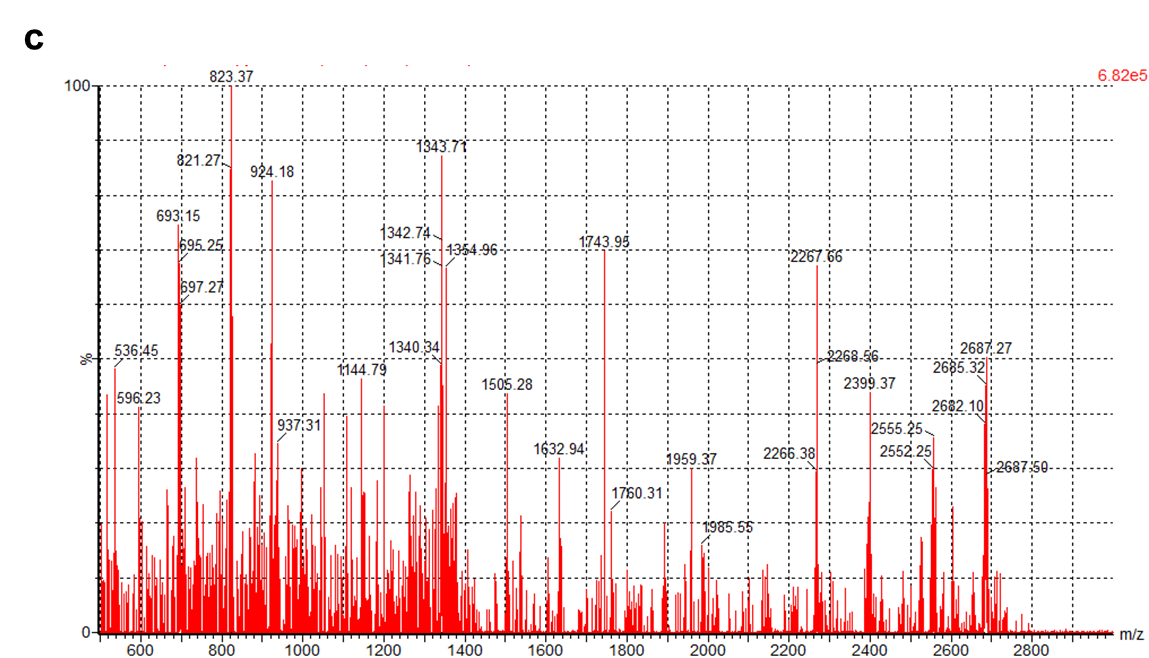


**Supplementary Fig. 13. UPLC-MS characterization of Pep-8.** (a) Structure of **Pep-8**. (b) UPLC characterization of **Pep-8** with the gradient of 5 - 95% CH_3_CN in H_2_O. (c) MS characterization of **Pep-8**. 2682.68 (Molecular Weight), 2682.10 (Found: [M]^+^).


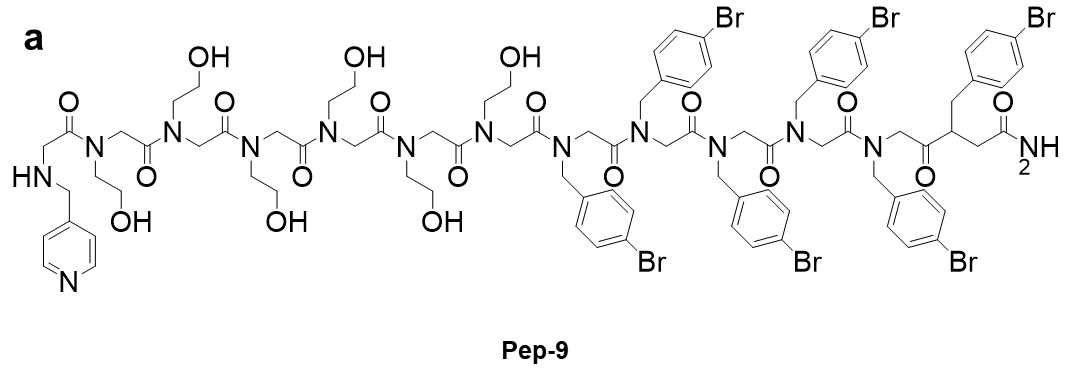


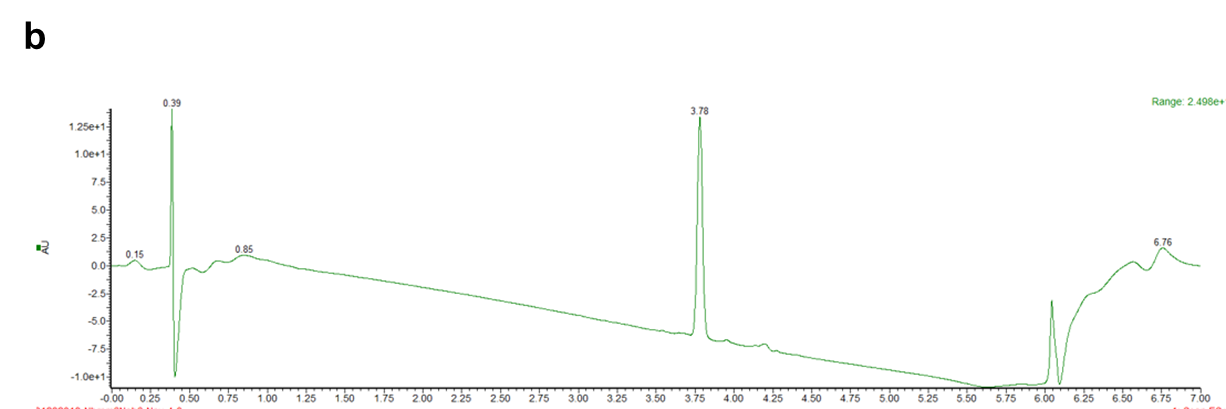


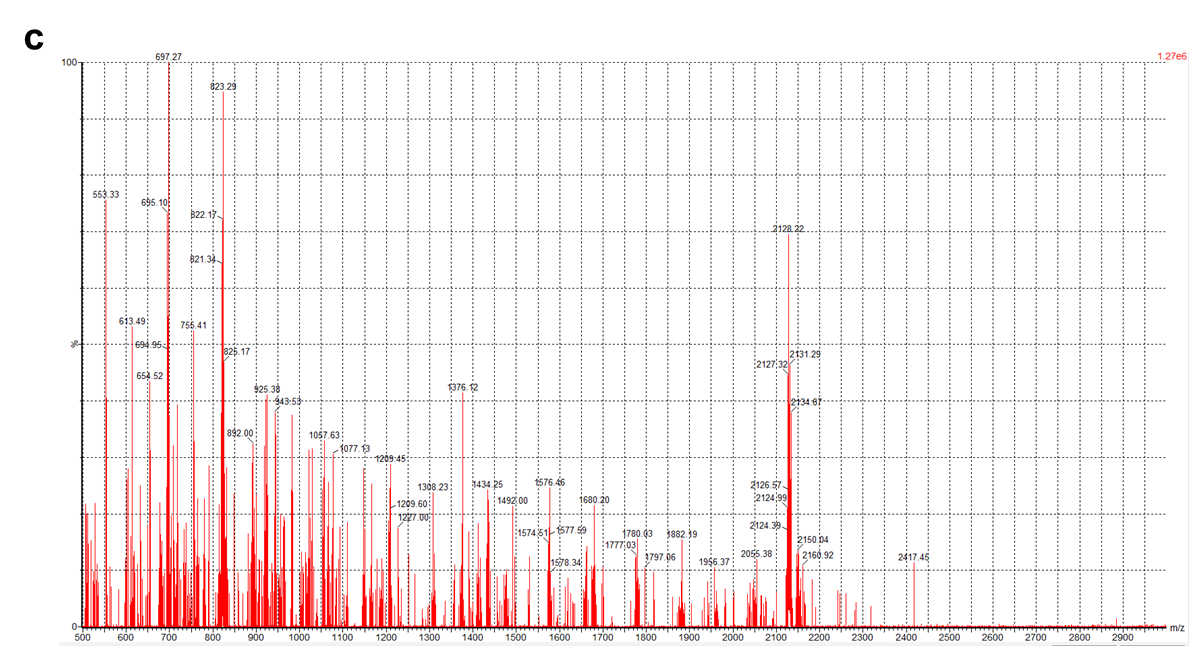


**Supplementary Fig. 14. UPLC-MS characterization of Pep-9.** (a) Structure of **Pep-9**. (b) UPLC characterization of **Pep-9** with the gradient of 5 - 95% CH_3_CN in H_2_O. (c) MS characterization of **Pep-9**. 2127.28 (Molecular Weight), 2128.22 (Found: [M+H]^+^).


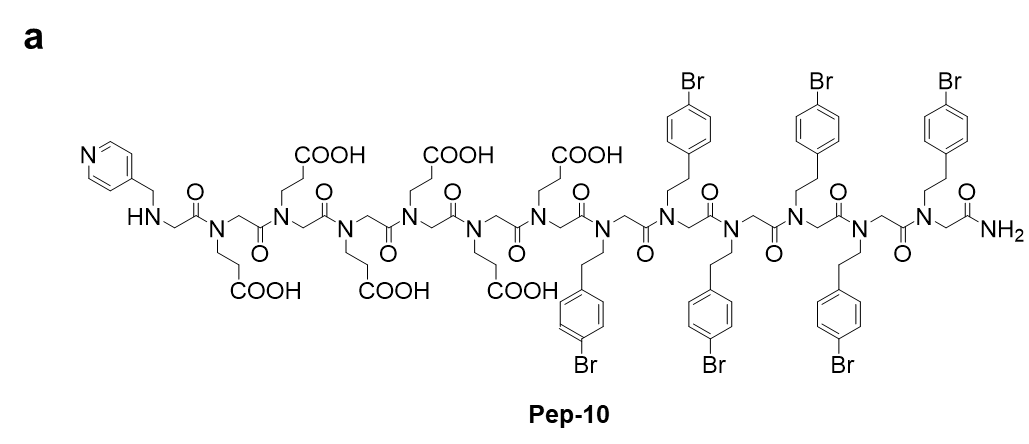


**b**


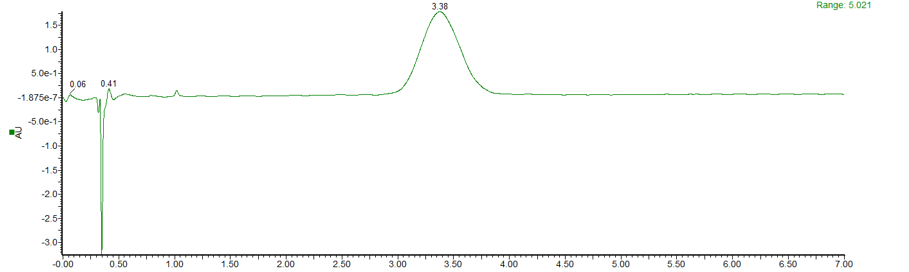


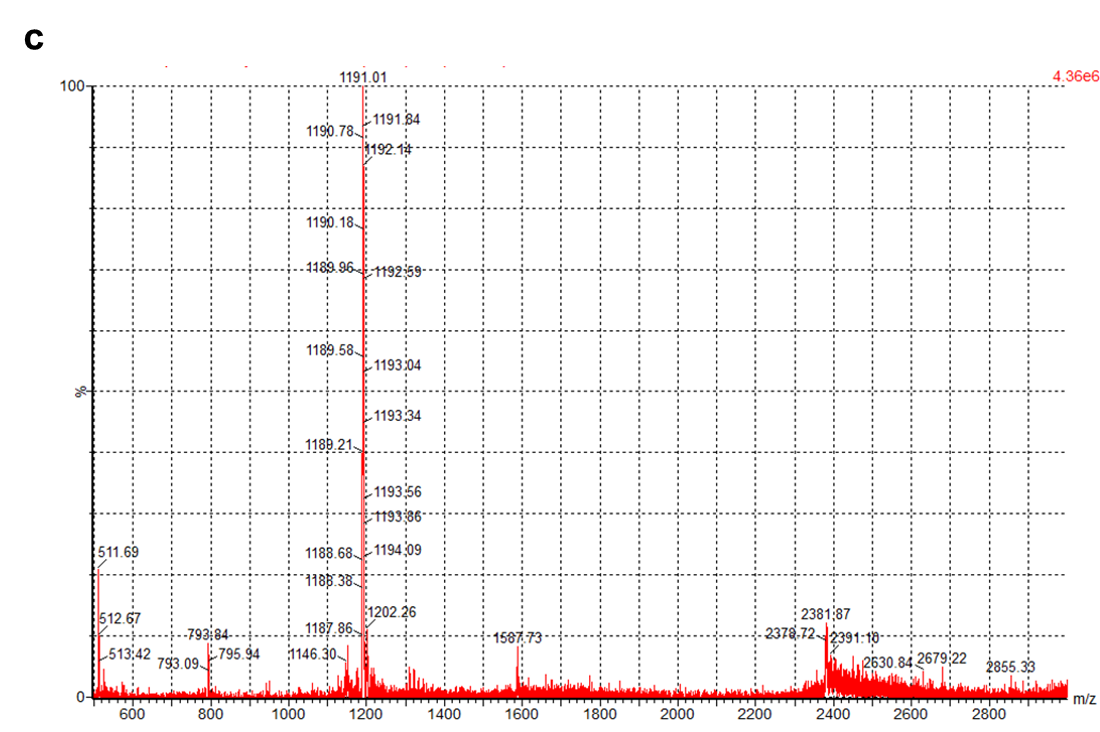


**Supplementary Fig. 15. UPLC-MS characterization of Pep-10.** (a) Structure of **Pep-10**. (b) UPLC characterization of **Pep-10** with the gradient of 40 - 80% CH_3_CN in H_2_O; (c) MS characterization of **Pep-10**. 2380.49 (Molecular Weight), 2381.87 (Found: [M+H] ^+^).


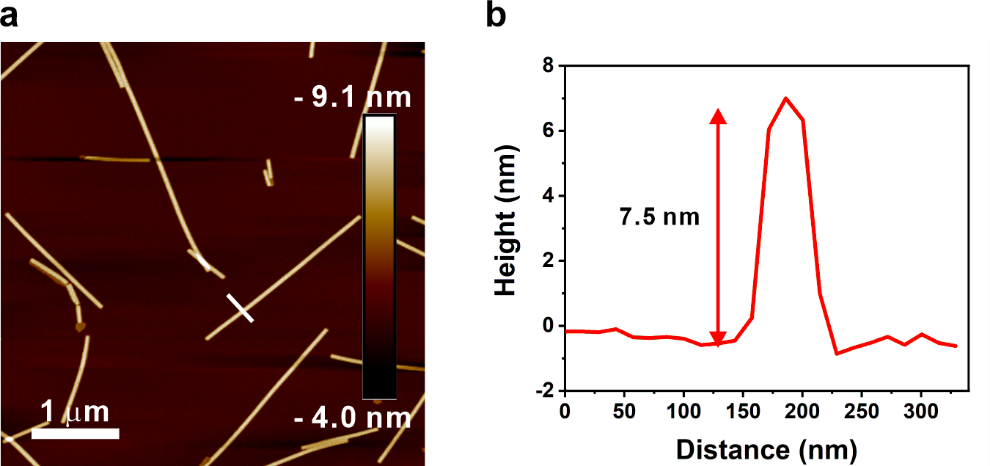


**Supplementary Fig. 16.** (a) AFM image of **Pep-2/hemin** nanotube and (b) height profile of a line scan in (a) showing the height of the nanotube is 7.5 nm.


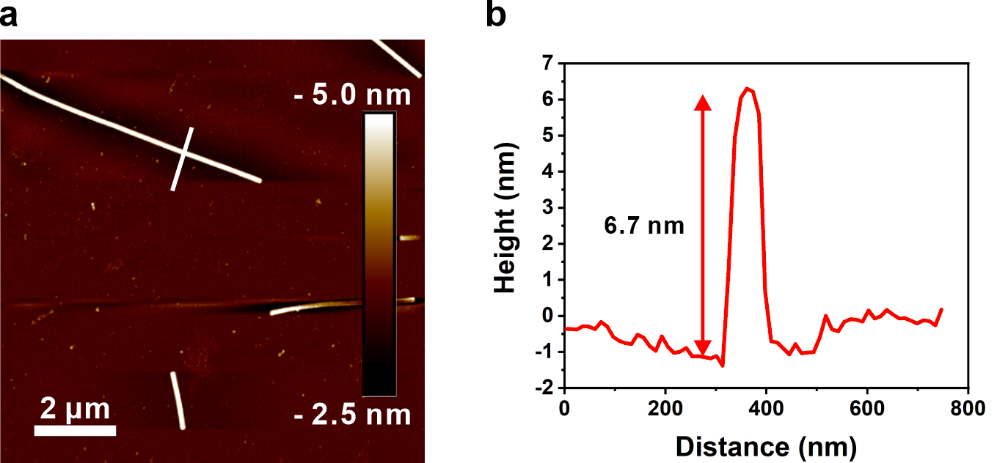


**Supplementary Fig. 17.** (a) AFM image of **Pep-3/hemin** nanotube and (b) height profile of a line scan in (a) showing the height of the nanotube is 6.7 nm.


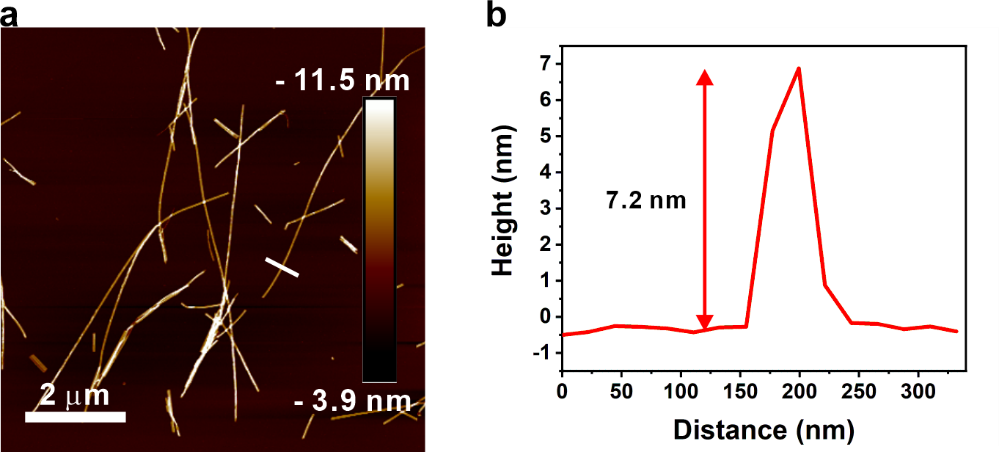


**Supplementary Fig. 18.** (a) AFM image of **Pep-4/hemin** nanotube and (b) height profile of a line scan in (a) showing the height of the nanotube is 7.2 nm.


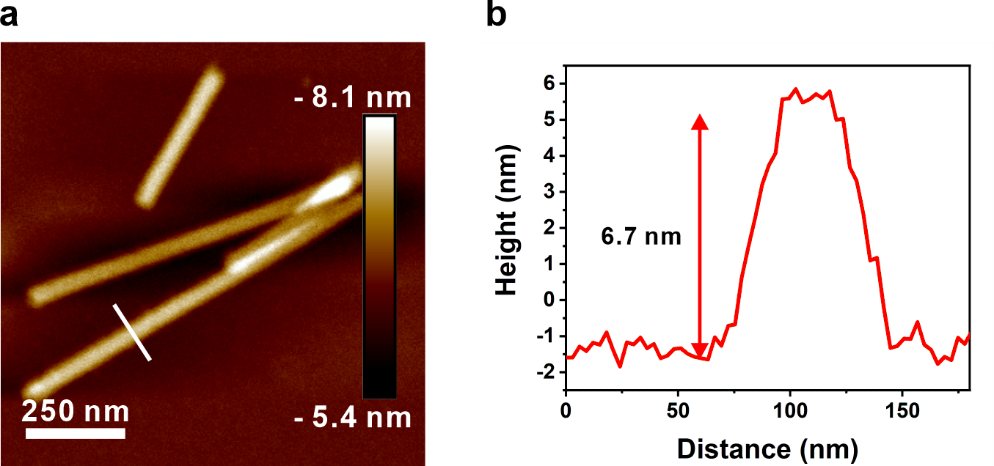


**Supplementary Fig. 19.** (a) AFM image of **Pep-5/hemin** nanotube and (b height profile of a line scan in (a) showing the height of the nanotube is 6.7 nm.


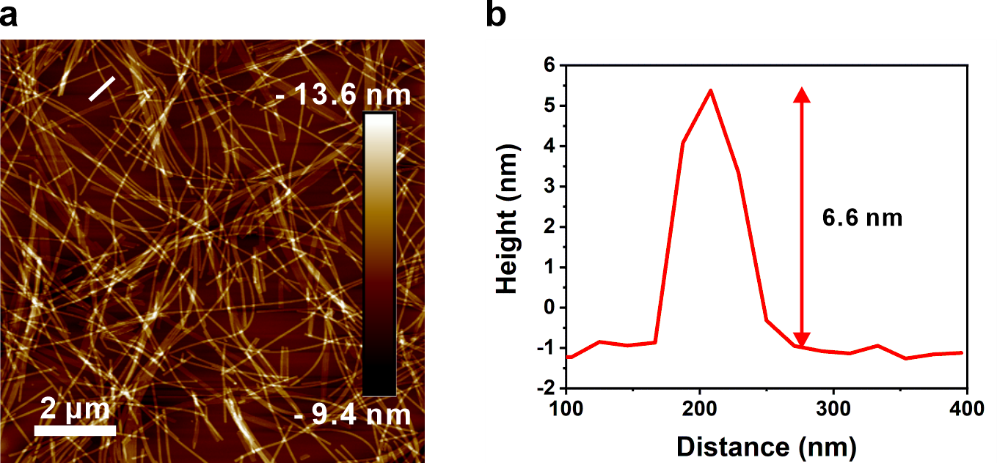


**Supplementary Fig. 20.** (a) AFM image of **Pep-6/hemin** nanotube and (b) height profile of a line scan in (a) showing the height of the nanotube is 6.6 nm.


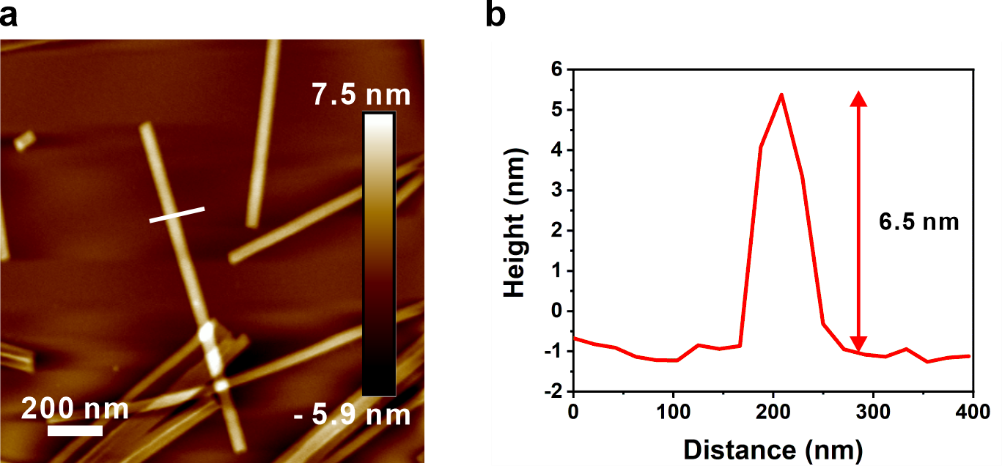


**Supplementary Fig. 21.** (a) AFM image of **Pep-7/hemin** nanotube and (b) height profile of a line scan in (a) showing the height of the nanotube is 6.5 nm.


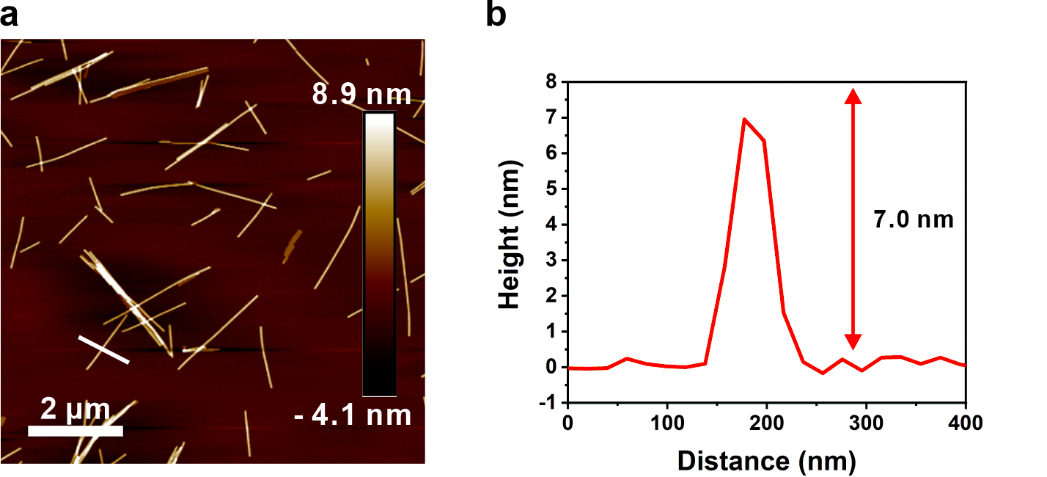


**Supplementary Fig. 22.** (a) AFM image of **Pep-8/hemin** nanotube and (b) height profile of a line scan in (a) showing the height of the nanotube is 7.0 nm.


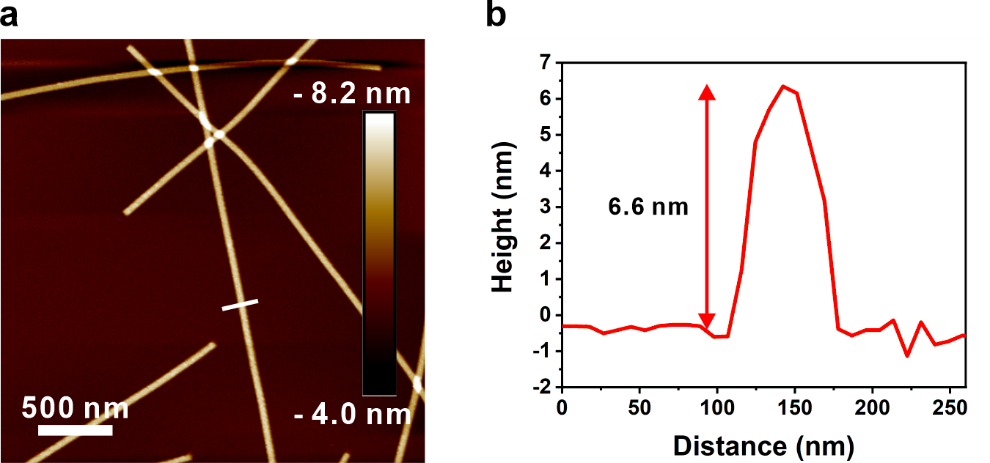


**Supplementary Fig. 23.** (a) AFM image of **Pep-9/hemin** nanotube and (b) height profile of a line scan in (a) showing the height of the nanotube is 6.6 nm.


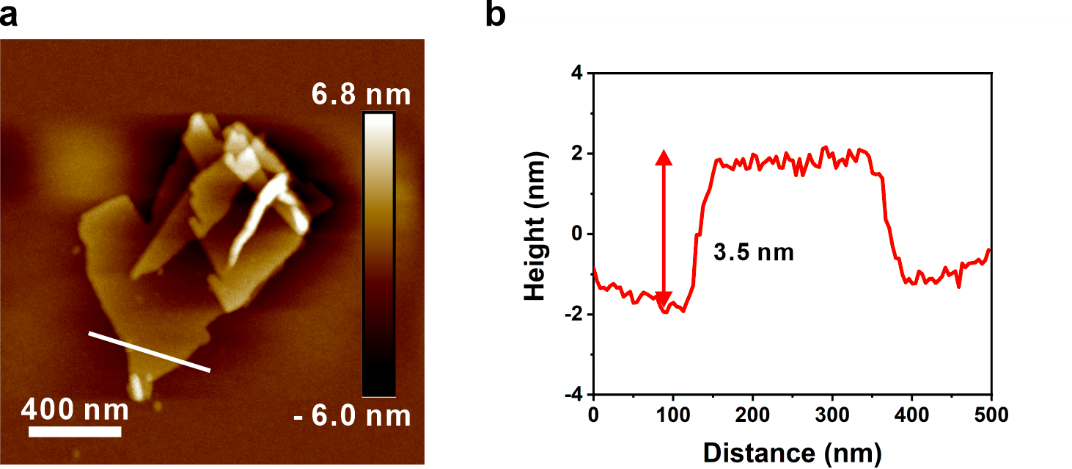


**Supplementary Fig. 24.** (a) AFM image of **Pep-10/hemin** nanosheet and (b) height profile of a line scan in (a) showing the height of the nanosheet is 3.5 nm.


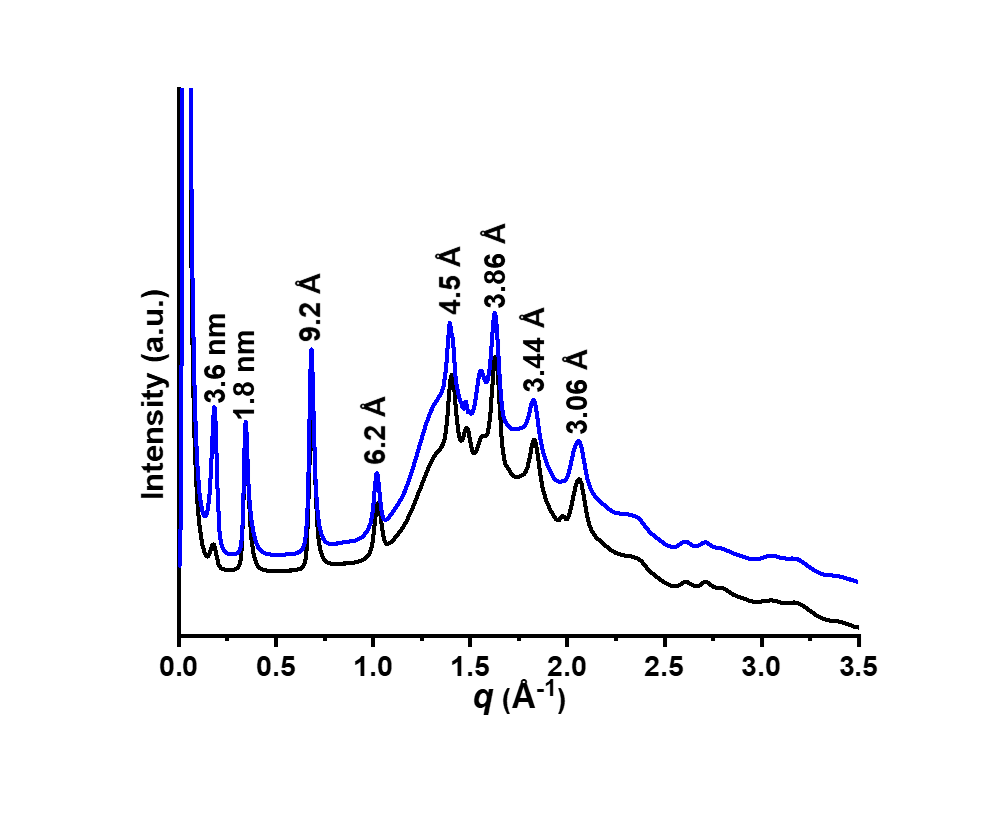


**Supplementary Fig. 25.** XRD data of **Pep-10/hemin** nanosheets (Black). All XRD peaks are similar to those of previously reported membrane-mimetic nanosheets (Blue),^1^ showing they have similar nanosheet structures.

**
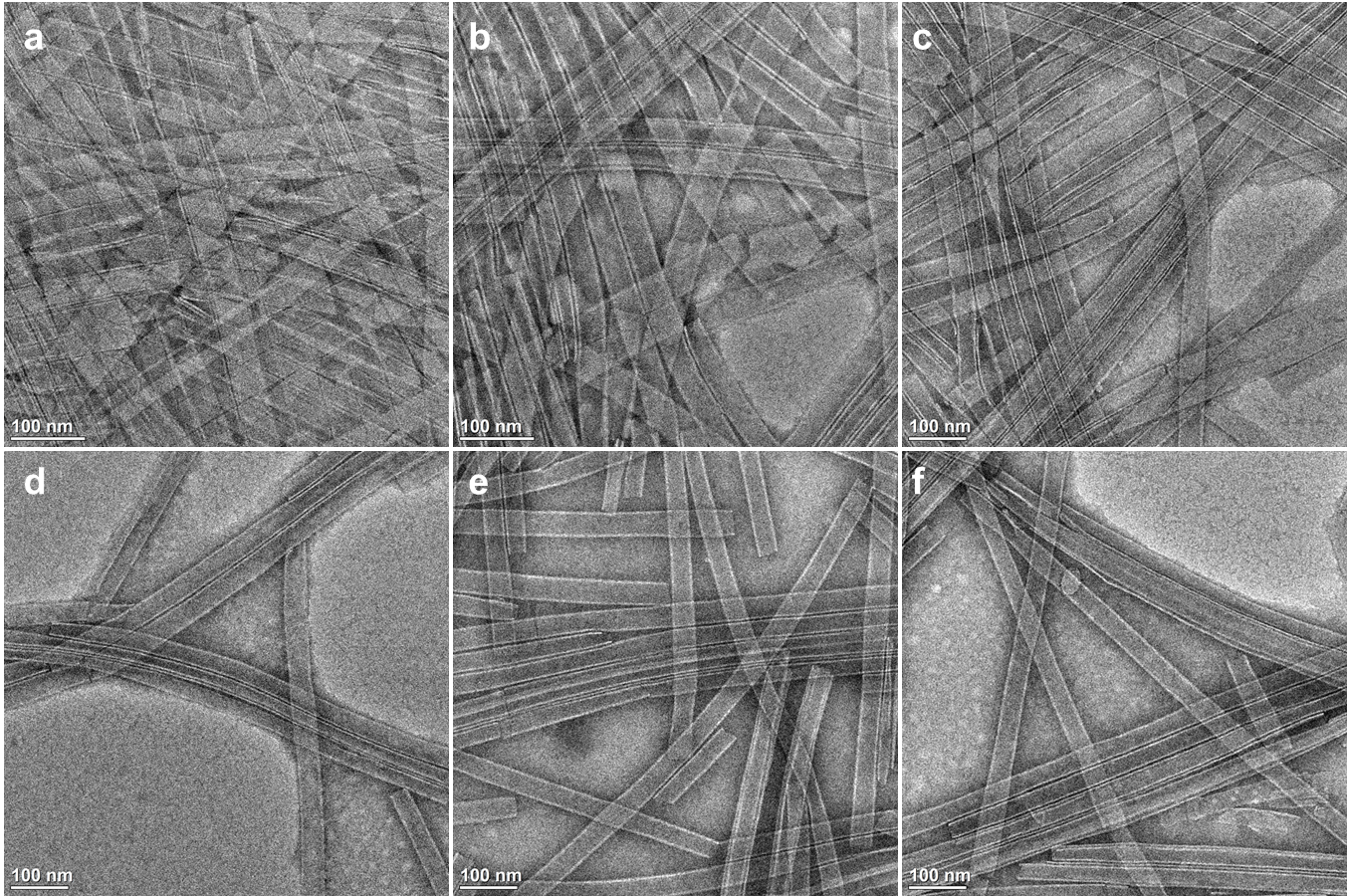
**

**Supplementary Fig. 26.** Negatively stained TEM images of Pep/hemin nanotubes after ABTS oxidation reactions under different temperatures. Reaction conditions: (a) **Pep-1/hemin**, 60 ºC, (b) **Pep-2/hemin**, 60 ºC, (c) **Pep-3/hemin**, 60 ºC, (d) **Pep-1/hemin**, 90 ºC, (e) **Pep-2/hemin**, 90 ºC, (f) **Pep-3/hemin**, 90 ºC.





**Supplementary Fig. 27.** Correlation between the slope values obtained in the four Lineweaver-Burk plots against inversed H_2_O_2_ concentration changes associated with Fig. 4a. The linear fitting shows a very small slope (0.00148) indicating parallel Lineweaver-Burk plots, and thus, the double replacement ping-pong mechanism. The error bar represents the standard deviation of three measurements.


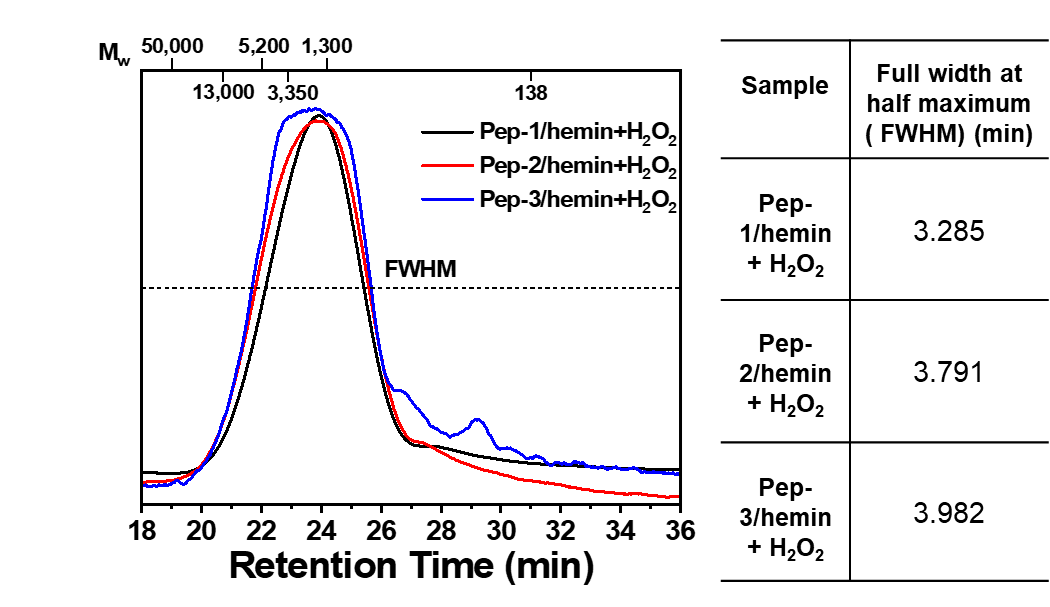


**Supplementary Fig. 28.** Gel permeation chromatography (GPC) profile of ethanol organosolv lignin (EOL) samples after treatment with **Pep-1/hemin**, **Pep-2/hemin,** or **Pep-3/hemin** in the presence of H­_2_O_2_. The full width at half maximum is used here to evaluate the relative dispersity of depolymerized EOL.


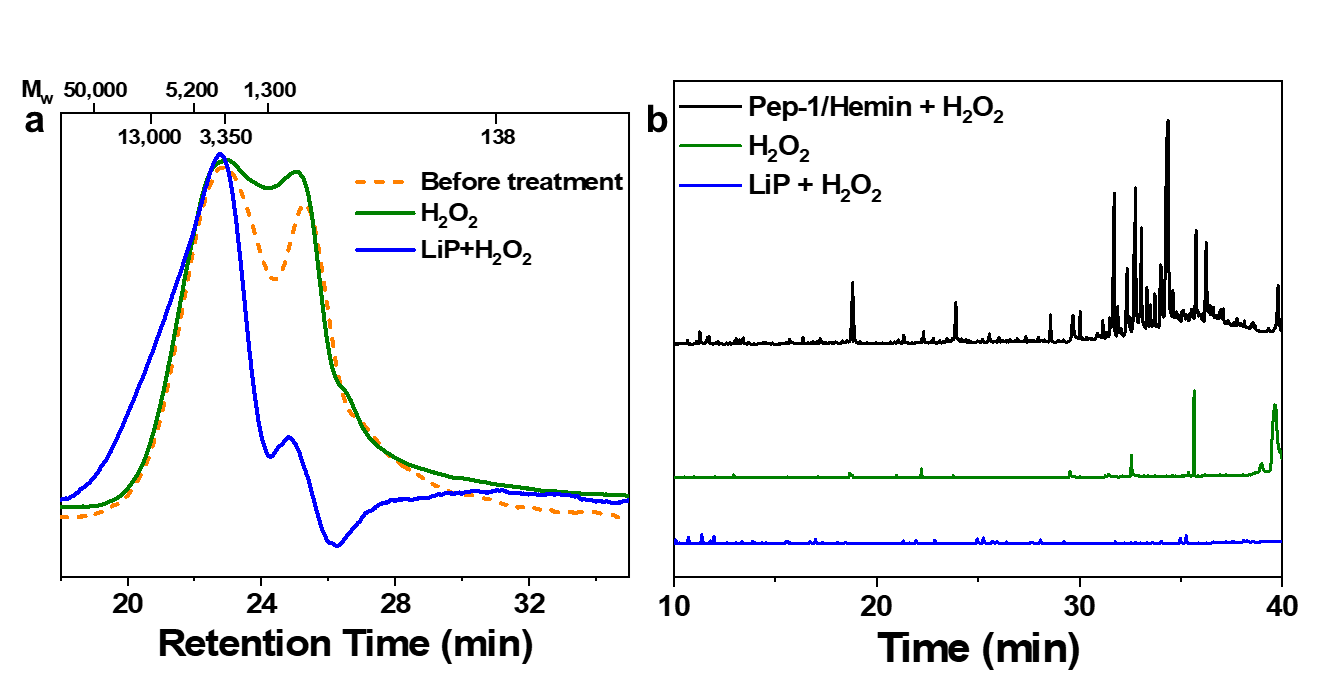


**Supplementary Fig. 29.** (a) Gel permeation chromatography (GPC) profiles of ethanol organosolv lignin (EOL) samples before treatment (orange), after treatment only with H_2_O_2_ (olive), or treatment with lignin peroxidase (LiP) in the presence of H­_2_O_2_ (blue). (b) Gas chromatography-mass spectroscopy (GC-MS) results of ethyl acetate extractable products from EOL samples treated with Pep-1/hemin (black), LiP (blue), or without catalysts (olive) at 60 ºC under acidic solution (pH ~4) in the presence of H_2_O_2_.

**Supplementary Table 1.** Mass balance obtained during the depolymerization of lignin with different conditions.

| **Sample** | **Yield of solid isolated after depolymerization (%)** | **Yield of phenolic compounds present in liquid after depolymerization (%)*** | **Yields of phenolic compounds from ethyl acetate extraction (%)**** | **Total amount (%)** |
| --- | --- | --- | --- | --- |
| 50 mg Lignin + **Pep-1/hemin** + H_2_O_2_ | 34.3 ± 0.36 | 61.7 | 59.1 ± 1.2 | 96.0 |
| 50 mg Lignin + **Pep-2/hemin** + H_2_O_2_ | 35.1 ± 0.52 | 60.4 | 57.1 ± 1.6 | 95.5 |
| 50 mg Lignin + **Pep-3/hemin** + H_2_O_2_ | 43.9 ± 1.54 | 47.2 | 45.3 ± 2.1 | 91.1 |
| 50 mg Lignin + H_2_O_2_ | 46.2 ± 0.92 | 10.2 | 12.3 ± 2.4 | 56.4 |

*****The yield of phenolic compound present in the solution after depolymerization was calculated based on the F-C analysis.^2^

****** The yield of the phenolic compound from ethyl acetate (EA) extraction of the corresponding solution after lignin depolymerization was calculated by dividing the mass of extracted compounds by the total lignin mass (50 mg).^2^ Calculation of the yield of EA extractable compounds was performed as follows: after lignin is depolymerized in an aqueous solution, water is removed by lyophilization and the dry weight (1) of the reaction mixture is obtained. EA is used to extract the phenolic compounds, and EA is removed under N_2_ flow, and the remaining mixture is weighed (2). Lignin residue was lyophilized and weighed (3). The yield of EA extractable compounds is calculated using this equation: Yield = Weight(2)/[Weight(1)-Weight(3)]*100%.

**Supplementary References:**

1 Ma, J. *et al.* Nanoparticle-Mediated Assembly of Peptoid Nanosheets Functionalized with Solid-Binding Proteins: Designing Heterostructures for Hierarchy. *Nano Lett.* **21**, 1636-1642, doi:10.1021/acs.nanolett.0c04285 (2021).

2 Ma, R. *et al.* Role of peracetic acid on the disruption of lignin packing structure and its consequence on lignin depolymerisation. *Green Chem.* **23**, 8468-8479, doi:10.1039/D1GC02300D (2021).
