# Supplementary material for: Highly stable and tunable peptoid/hemin enzymatic mimetics with natural peroxidase-like activities
Source: Nat Commun. 2022 May 31;13:3025. doi: 10.1038/s41467-022-30285-9 (PMC9156750; doi:10.1038/s41467-022-30285-9)
Supplement: Supplementary file 2 — Description of Additional Supplementary Information [file 41467_2022_30285_MOESM2_ESM.pdf]

List of supplementary materials:

Supplementary Fig. 1. TEM images of Pep-1/hemin nanotube assembly.
Supplementary Fig. 2. UV-Vis spectra of the mixture of hemin and Pep-1 at different molar ratios.
Supplementary Fig. 3. AFM images of Pep-1/Cu, Pep-1/Mn, and Pep-1+hemin assemblies.
Supplementary Fig. 4. XRD patterns of Pep-1/Cu, Pep-1/Mn, and Pep-1/hemin nanotubes.
Supplementary Fig. 5. SEM images and EDS measurements of assembled nanotube catalysts.
Supplementary Fig. 6 to Supplementary Fig. 15. UPLC-MS characterization of Pep-1 to Pep-10.
Supplementary Fig. 16 to Supplementary Fig. 23. AFM images of Pep/hemin nanotubes and their corresponding height profiles.
Supplementary Fig. 24. AFM images of Pep-10/hemin nanosheet and its height profile.
Supplementary Fig. 25. XRD data of Pep-10/hemin nanosheet.
Supplementary Fig. 26. TEM images of Pep/hemin nanotubes after ABTS oxidation reactions.
Supplementary Fig. 27. Correlation between the slope values obtained in the four Lineweaver-Burk plots against inversed H_2O_2 concentration changes associated with Fig. 4a.
Supplementary Fig. 28. Gel permeation chromatography (GPC) profile of ethanol organosolv lignin (EOL) samples after treatment with Pep-1/hemin, Pep-2/hemin, or Pep-3/hemin in the presence of H_2O_2.
Supplementary Fig. 29. GPC profiles of ethanol organosolv lignin (EOL) samples before and after treatments, and GC-MS results of ethyl acetate extractable products from treated EOL samples.
Supplementary Table S1. Mass balance obtained during the depolymerization of lignin with different conditions.
